# Protection Activity of 1,4-Naphthoquinones in Rotenone-Induced Models of Neurotoxicity

**DOI:** 10.3390/md22020062

**Published:** 2024-01-25

**Authors:** Irina Agafonova, Ekaterina Chingizova, Elena Chaikina, Ekaterina Menchinskaya, Sergey Kozlovskiy, Galina Likhatskaya, Yuri Sabutski, Sergey Polonik, Dmitry Aminin, Evgeny Pislyagin

**Affiliations:** 1 G.B. Elyakov Pacific Institute of Bioorganic Chemistry, Far-Eastern Branch of the Russian Academy of Science, 690022 Vladivostok, Russia; agafonova@piboc.dvo.ru (I.A.); martyyas@mail.ru (E.C.); chaykin.dima@yandex.ru (E.C.); ekaterinamenchinskaya@gmail.com (E.M.); sergeimerx@gmail.com (S.K.); galin@piboc.dvo.ru (G.L.); alixar2006@gmail.com (Y.S.); sergpol@piboc.dvo.ru (S.P.); daminin@piboc.dvo.ru (D.A.); 2 Department of Biomedical Science and Environmental Biology, Kaohsiung Medical University, No. 100, Shin-Chuan 1st Road, Sanmin District, Kaohsiung City 80708, Taiwan

**Keywords:** 1,4-naphthoquinones, neuronal cells, macrophages, rotenone, neuroprotection, Parkinson’s disease

## Abstract

The MTS cell viability test was used to screen a mini library of natural and synthetic 1,4-naphthoquinone derivatives (1,4-NQs) from marine sources. This screening identified two highly effective compounds, **U-443** and **U-573**, which showed potential in protecting Neuro-2a neuroblastoma cells from the toxic effects of rotenone in an in vitro model of neurotoxicity. The selected 1,4-NQs demonstrated the capability to reduce oxidative stress by decreasing the levels of reactive oxygen species (ROS) and nitric oxide (NO) in Neuro-2a neuroblastoma cells and RAW 264.7 macrophage cells and displayed significant antioxidant properties in mouse brain homogenate. Normal mitochondrial function was restored and the mitochondrial membrane potential was also regained by 1,4-NQs after exposure to neurotoxins. Furthermore, at low concentrations, these compounds were found to significantly reduce levels of proinflammatory cytokines TNF and IL-1β and notably inhibit the activity of cyclooxygenase-2 (COX-2) in RAW 264.7 macrophages. The results of docking studies showed that the 1,4-NQs were bound to the active site of COX-2, analogically to a known inhibitor of this enzyme, SC-558. Both substances significantly improved the behavioral changes in female CD1 mice with rotenone-induced early stage of Parkinson’s disease (PD) in vivo. It is proposed that the 1,4-NQs, **U-443** and **U-573**, can protect neurons and microglia through their potent anti-ROS and anti-inflammatory activities.

## 1. Introduction

Parkinson’s disease (PD) is an age-dependent and gradually progressive neurodegenerative disorder of great societal importance, ranking second only to Alzheimer’s disease (AD) in terms of its frequency in occurrence. PD is distinguished by a progressive degeneration of dopaminergic neurons within the nigrostriatal system of the brain, which leads to impairments primarily of motor function—resting tremor, hypokinesia, muscle rigidity, and postural instability—accompanied by nonmotor symptoms such as cognitive impairment and autonomic dysfunction [1,2,3]. It is assumed that the etiology of PD is associated with genomic, epigenetic, and environmental factors leading to conformational changes and deposits in the cytoplasm of neurons of protein aggregates (Lewy bodies) due to abnormalities in the ubiquitin–proteasome system, impaired differentiation of dopaminergic neurons, and dysregulation of function and oxidative stress, which ultimately causes neuronal death [4].

Recent studies have indicated the direct involvement of activated microglia in the processes of degeneration of dopaminergic neurons in the substantia nigra, specifically through the release of proinflammatory agents. The results revealed that the inflammation of microglia, triggered by bacterial lipopolysaccharide (LPS), causes excessive production of IL-1β, IL-6, IL-8, and TNF, along with the activation of inducible nitric oxide synthase (iNOS) [5]. This leads to elevated levels of reactive oxygen species (ROS) and nitric oxide (NO), which have detrimental effects on the normal functioning and essential activity of neurons [6,7].

Rotenone, an isoflavonoid, occurs naturally in various parts of Leguminosa plants and is utilized as a widely effective insecticide and pesticide. In experimental models of Parkinson’s disease (PD), both in in vitro and in animals, rotenone, a neurotoxic compound, is commonly employed as a trigger to induce neurodegenerative changes. Rotenone can impede the flow of electrons in the mitochondrial respiratory chain by inhibiting the electron transfer and interrupting the movement of electrons from the iron–sulfur cluster in complex I to ubiquinone. Due to this process, an excess of electrons accumulates in the mitochondrial matrix, specifically as NADPH. This surplus of electrons results in the conversion of oxygen into ROS, which can cause harm to mitochondrial components and DNA. In addition to its impact on mitochondrial respiration, rotenone also obstructs the formation of microtubules from tubulin. Consequently, these effects lead to neuronal dysfunction and eventual cell death [8,9].

1,4-Naphthoquinones (1,4-NQs) play a special role in animals, including marine organisms, and plants as their metabolites. There is a broad spectrum of cytoprotective properties exhibited by compounds, which originate from natural sources as well as through synthetic modifications. It has been established that these compounds possess a diverse range of biological activities, including antifungal, antibacterial, antioxidant, cardioprotective, hepatoprotective, anti-ischemic, and various other properties. There is an increasing number of scientific papers exploring the protective role of 1,4-naphthoquinones in disorders characterized by the degeneration of neurons such as AD and PD [10,11,12,13,14,15,16,17,18,19].

In recent studies, we successfully synthesized a mini library of 5,8-dihydroxy-1,4-naphthoquinone derivatives (consisting of 44 compounds) derived from the natural pigments found in echinoderms. We evaluated the cytotoxic and neuroprotective effects of these compounds against mouse neuronal Neuro-2a cells in two in vitro models of PD, induced by paraquat (PQ) and 6-hydroxydopamine (6-OHDA) neurotoxins. Some compounds exhibiting pronounced neuroprotective properties [20,21,22] were selected for future examinations. According to the published data, the most active studied 1,4-NQ derivatives at low concentrations were found to effectively defend cell biomembranes, normalize the cell cycle, suppress oxidative stress, normalize the mitochondrial function altered by neurotoxins, and eliminate induced inflammation. 

In this study, we examined the protective effects of the two most potent 1,4-NQ derivatives, namely **U-443** and **U-573**, demonstrating in preliminary in vitro experiments a significant ability to preserve the viability of neuronal cells in the presence of PQ, 6-OHDA, and rotenone, in both cellular and animal models of parkinsonism.

For this purpose, we used two cell cultures, Neuro-2a mouse neuroblastoma cells and RAW 264.7 mouse macrophage cells, mimicking brain neurons and microglia, respectively. Rotenone was used as a PD inducer and LPS was used to induce microglial inflammation. We evaluated the potential of the two selected 1,4-NQs to reduce the generation of ROS and NO in cells subjected to neurotoxin and LPS treatment. Additionally, we examined their impact on mitochondrial membrane potential (MMP), their influence on the production of proinflammatory cytokines such as TNF and IL-1β, their ability to inhibit the activity of cyclooxygenase 2 (COX-2) in macrophages, and their antioxidant activity in mouse brain homogenate. Furthermore, the effect of the two 1,4-naphthoquinones on spontaneous locomotion in an in vivo murine rotenone-induced early stage of the PD model was investigated.

## 2. Results

### 2.1. Synthesis of 6-Ethyl-2,3,5,8-tetrahydroxy-1,4-naphthoquinone (ethylspinazarin) and 2-(Tetra-O-acetyl- β-d-glucopyranosyloxy)-3-ethyl-5,8-dihydroxy-1,4-naphthoquinone

In order to determine suitable inhibitors for in vivo experiments targeting PD, we conducted a screening of a limited collection comprising both natural and synthetic 1,4-naphthoquinones and their O- and S-glucoside derivatives [23]. This library consisted of various compounds, among which we focused on two specific ones: 6-ethyl-2,3,5,8-tetrahydroxy-1,4-naphthoquinone, **U-573** (the minor sea urchin pigment (ethylspinazarin) of *Scaphechinus mirabilis*) and the synthetic acetylated 1,4-naphthoquinone-*O*-glucoside **U-443** of ophiuroid naphthazarine pigment **6**.

**Scheme 1:**
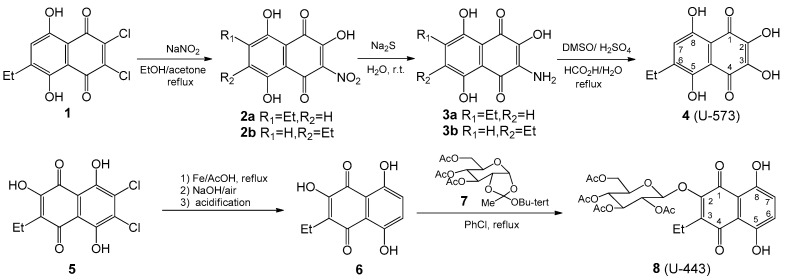
Synthesis of ethylspinazarin **4** (**U-573**) and acetylated 1,4-naphthoquinone-*O*-glucoside **8** (**U-443**).

Ethylspinazarin **4** (**U-573**) is readily prepared by conversion of the available ethyldichloroNQ **1** mixture of two isomeric nitroNQs **2a,b**, subsequent reduction of nitroNQs with sodium sulfide in aminoNQs **3a,b** in water solution and acid-catalyzed transformation of aminoNQs in ethylspinazarin **4** (**U-573**) under DMSO action in water-formic acid solution at reflux (Scheme 1) [24,25]. The total yield of ethylspinazarin **4** (**U-573**) in the three stages was 62%.

**Figure 1:**
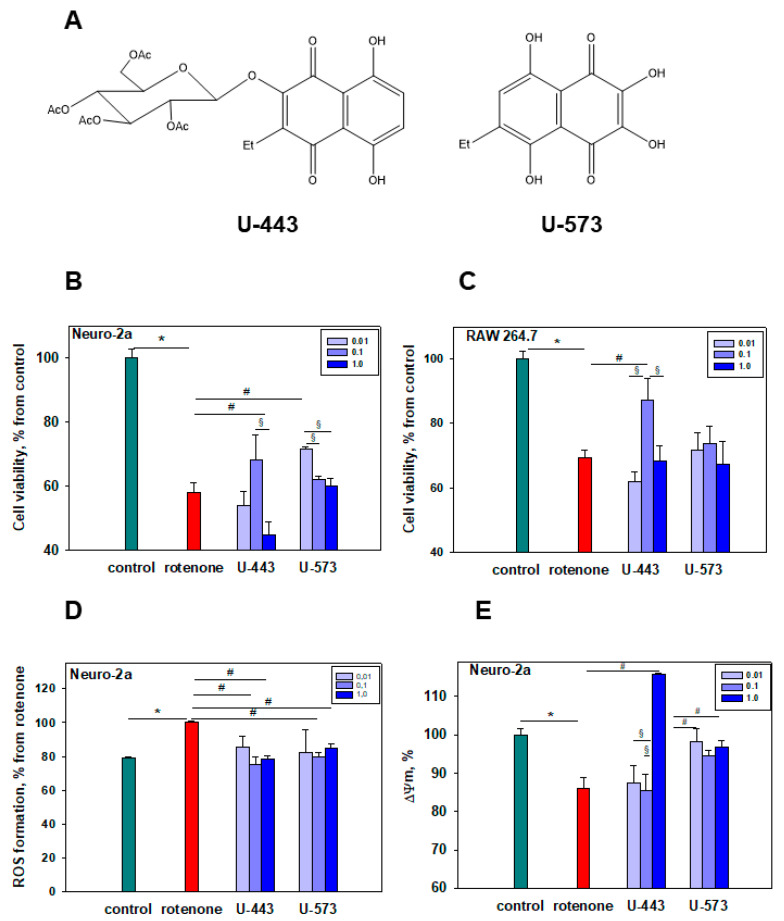
Protective effects of 1,4-NQs against rotenone-induced neurotoxicity. (**A**) The chemical structures of **U-443** (left) and **U-573** (right) are displayed. The impact of 1,4-NQs, specifically **U-443** and **U-573**, on the viability of Neuro-2a (**B**) and RAW 264.7 (**C**) cells is quantified. In these experiments, cells were exposed to rotenone at a concentration of 10 µM, and cell viability was assessed using the MTS method. The effects of 1,4-NQs on ROS production (**D**) and mitochondrial membrane potential (**E**) in Neuro-2a cells were evaluated. ROS intracellular levels and MMP values were measured using spectrofluorimetry with H2DCF-DA and TMRM fluorescent probes, respectively. The cell concentration used was 3 × 10^4^ cells/well. The inserts in the figure indicate the color codes that correspond to the concentrations of the tested compounds in µM. The data presented are depicted as mean ± SEM (n = 3). * *p* < 0.05 compared with control cells; ^#^ *p* < 0.05 compared with cells exposed to rotenone alone; ^§^ *p* < 0.05 denotes significant differences between cells exposed to different concentrations of each compound.

Acetylglucoside **8** (**U-443**) was synthesized by autocatalytic condensation of 2,5,8-trihydroxy-3-ethyl-1,4-naphthoquinone **6** with 3,4,6-tri-*O*-acetyl-α-d-glucopyranose 1,2-(tert-butoxy orthoacetate) **7** [26]. Starting NQ **6** was obtained by dechlorination of the available dichloroNQ **5** through Fe/AcOH treatment and air oxidation of the reaction mixture in one pot [27]. The synthesis and chemical structures of the studied compounds **U-443** and **U-573** are shown in Scheme 1 and Figure 1A.

### 2.2. The Evaluation of the Protective Effects of 1,4-NQs in an In Vitro Model of PD Induced by Rotenone

#### 2.2.1. Cytoprotective Properties of 1,4-NQs in MTS Test

In order to assess the protective effects of 1,4-NQ derivatives in a rotenone-induced PD model using mouse neuroblastoma Neuro-2a cells, a neurotoxin was added into the culture medium at a concentration of 10 µM, resulting in roughly 40–50% cell death. Following the analysis of the mini library comprising 44 1,4-NQs, two compounds (**U-443** and **U-573**) demonstrated significant effectiveness in protecting cells against the detrimental effects of neurotoxins. These compounds were chosen for further investigation based on their promising results. During our previous investigation [21], we determined the cytotoxic activity (EC_50_) of **U-443** and **U-573** on mouse neuronal Neuro-2a cells. The EC_50_ values were found to be 4.46 µM for **U-443** and >100 µM for **U-573**. The cell suspension was treated with these compounds at noncytotoxic concentrations ranging from 0.01 to 1.0 µM, one hour prior to the application of the PD inducer. The cells were then incubated for one day, and thereafter, the viability of the cells was assessed.

The results showed that both quinones exhibited a significant increase in Neuro-2a cell viability when exposed to the neurotoxin, resulting in approximately a 20% increase compared with cells treated with rotenone alone. When administered at a concentration of 0.1 µM, **U-443** effectively protected neuronal cells from the damaging effects of rotenone, resulting in a notable 17.7% increase in the number of viable cells. Similarly, at a concentration of 0.01 µM, substance **U-573** exhibited a 23.3% increase in this parameter compared with control cells exposed to rotenone only. Although the protection provided by the 1,4-NQs was evident, it should be noted that it was only partial. The values of cell viability in the presence of rotenone and the test compounds were found to be significantly different from those of intact cells. The comparison of the effects of different concentrations showed that the effect of **U-573** (0.01 µM) significantly differs from the concentrations of 0.1 and 1.0 µM. At the same time, **U-443** at a concentration of 1.0 µM markedly reduced the cell viability to a level lower than that of rotenone alone (Figure 1B). The dose–effect curves for **U-443** and **U-573** cytotoxic effects are shown in Figure 4A.

The impact of rotenone on the viability of RAW 264.7 cells yielded similar outcomes. At a concentration of 10 µM, rotenone significantly decreased cell viability by approximately 30%. However, the compounds **U-443** and **U-573** exhibited protective effects on macrophage cell viability. Among them, **U-443** proved to be the most potent, elevating cell viability to nearly baseline levels at a concentration of 0.1 µM (*p* < 0.05 compared with intact cells). Notably, this effect showed significant variations (*p* < 0.05) when compared with the ineffective concentrations of 0.01 and 1.0 µM. On the other hand, **U-573** at concentrations of 0.01 µM and 0.1 µM exhibited insignificant cell protection ranging from approximately 3.4% to 6.2%. No significant differences were observed between the effects of the examined concentrations of **U-573** (Figure 1C).

#### 2.2.2. Evaluation of Rotenone-Induced ROS Production and MMP in Neuro-2a Cells in the Presence of 1,4-NQs

The suppressive effects of 1,4-NQs **U-443** and **U-573** on the oxidative burst induced by rotenone in neuronal cells were investigated. To achieve this, the compounds were incubated with the cells for 1 h at noncytotoxic concentrations before the introduction of rotenone into the culture. Subsequently, the cells were incubated for 1 h with rotenone at a concentration of 10 µM. Afterward, the levels of ROS and mitochondrial membrane potential (MMP) were determined using spectrofluorimetry.

The administration of rotenone led to a noticeable amplification of ROS production and caused a disruption in the MMP in neuronal cells. However, when the compounds **U-443** and **U-573** were present at concentrations of 0.1 and 1.0 μM, they significantly reduced the formation of ROS in cells exposed to rotenone. The most potent effect was observed at a concentration of 0.1 μM. **U-443** and **U-573** decreased the ROS levels in cells by 24.5% and 20.2%, respectively (Figure 1D). The protection exerted by these compounds was complete as there were no significant differences (*p* > 0.05) in ROS levels between cells incubated with rotenone and substances, and intact control cells.

An increase in MMP values under rotenone action means depolarization of mitochondrial potential, which leads to mitochondrial dysfunction and precedes cell apoptosis. The tested 1,4-NQs showed the ability to prevent depolarization and restore MMP to nearly normal levels. **U-443** was not effective at concentrations of 0.01 and 0.1 µM, but at a concentration of 1.0 µM, it significantly increased the MMP values compared with the effect of rotenone alone. This compound caused a slight hyperpolarization, indicated by increased TMRM probe fluorescence, compared with the control cells that were not exposed to the neurotoxin. On the other hand, **U-573** at concentrations of 0.01 and 1.0 µM successfully prevented depolarization and restored the MMP of neuronal cells almost to baseline values. There were no significant differences (*p* > 0.05) compared with the intact control cells between all studied concentrations of **U-573** (Figure 1E).

### 2.3. Assessing the Anti-Inflammatory Characteristics of 1,4-NQs Using an In Vitro Model of Inflammation

#### 2.3.1. Determination of ROS and the NO Production Levels in RAW 264.7 cells Caused by LPS or Rotenone in the Presence of 1,4-NQs

To assess the anti-inflammatory properties of the 1,4-NQs in an inflammation model with mouse macrophage RAW 264.7 cells, the cell culture medium was supplemented with bacterial LPS at a concentration of 1.0 µg/mL and then incubated at 37 °C for 24 h. Prior to the LPS treatment, the compounds **U-443** and **U-573** were added at concentrations ranging from 0.01 to 1.0 µM and allowed to incubate for one hour. Subsequently, spectrofluorimetric measurements were used to determine the levels of ROS and NO in the cells.

**Figure 2:**
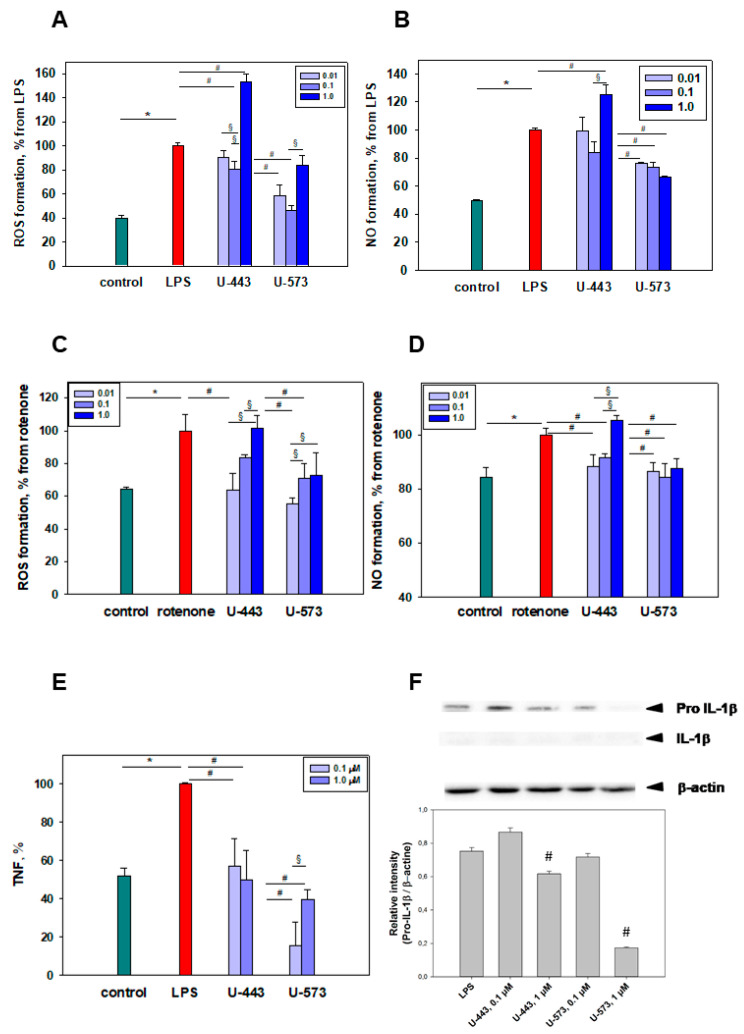
The impact of 1,4-NQs on the levels of ROS (**A**), NO (**B**), TNF (**E**), and IL-1β (**F**) production in RAW 264.7 cells treated with LPS (1.0 µg/mL), as well as the levels of ROS (**C**) and NO (**D**) in macrophages treated with rotenone (10 µM). ROS and intracellular NO levels were measured by spectrofluorimetry using H2DCF-DA and DAF-FM fluorescent probes. TNF levels were determined using ELISA, while IL-1β production was detected via immunoblotting. The concentration of cells per well was 3 × 10^4^. The inserted color codes correspond to the concentrations of the tested compounds in µM. The data are presented as mean ± SEM (n = 3). Statistical significance is denoted as * *p* < 0.05 compared with control cells, ^#^ *p* < 0.05 compared with cells exposed to the inducer (rotenone or LPS) alone, and ^§^ *p* < 0.05 compared between cells exposed to different concentrations of each compound.

During cell cultivation in the presence of LPS, the studied 1,4-naphthoquinones suppressed ROS production amplified by bacterial lipopolysaccharide in RAW 264.7 cells. Figure 2A demonstrates the ability of **U-443** and **U-573** compounds at concentrations ranging from 0.01 to 0.1 μM to decrease ROS production in macrophages. The most active was **U-573**; this compound significantly suppressed the formation of ROS by 43.1–54.4% of the control with LPS, while **U-443** in the same concentration range reduced the level of ROS by 11.8–20.9%. Only **U-573** at a concentration of 0.1 μM exhibited the capability to decrease ROS levels to the baseline level. This reduction was statistically insignificant (*p* > 0.05) compared with the control intact cells. No significant differences were found between different compound concentrations except 1.0 μM. Neither substance at a concentration of 1.0 μM decreased the ROS level in macrophages, and **U-443** at this concentration even increased ROS levels in comparison with LPS alone.

The selected 1,4-NQs, when preincubated with RAW 264.7 cells, showed a reduction in NO production in cells exposed to LPS. The most significant impact was observed with **U-573** at a concentration of 1.0 μM, resulting in a reduction of approximately 27.5%. Substance **U-443** displayed no effectiveness at any of the studied concentrations, and intriguingly, it even caused an increase in intracellular NO levels when used at a concentration of 1.0 μM compared with LPS alone. Neither substance in all studied concentrations decreased the NO amount in macrophages up to the baseline level (Figure 2B).

As depicted in Figure 2C,D, the neurotoxin rotenone induced a substantial increase in ROS and NO production in RAW 264.7 cells. Notably, both substances, **U-443** and **U-573**, exhibited a significant reduction in ROS formation in cells exposed to rotenone. A dose-dependent effect was observed for **U-443** and **U-573** within the concentration range of 0.01–1.0 µM. At a concentration of 0.01 µM, these compounds exerted the most potent effect, leading to a significant reduction in ROS levels by 30.5% and 42.9%, respectively (Figure 2C). This protection was almost complete as it was statistically insignificant (*p* > 0.05) when compared with the ROS levels in intact control cells.

Preincubation of macrophage cells with **U-443** and **U-573** decreased the NO amount in rotenone-exposed cells. Both compounds at concentrations of 0.01 µM and 0.1 µM reduced intracellular NO levels by 11.7% and 15.7%, respectively. This protection was almost full (*p* < 0.05 compared with NO level in control intact cells). There was no significant dose response in the concentration range of 0.01–0.1 µM for **U-443** and 0.01–1.0 µM for **U-573**. Compound **U-443** at a concentration of 1.0 µM caused a slight elevation in intracellular NO levels in cells treated with rotenone (Figure 2D).

#### 2.3.2. Evaluation of Proinflammatory Cytokine Production Levels in RAW 264.7 Cells Caused by LPS in the Presence of 1,4-NQs

The effect of 1,4-NQs on cytokine TNF production in RAW 264.7 macrophages was evaluated by ELISA. For this purpose, macrophage cells were incubated in 96-well plates in the presence of LPS (1 µg/mL) and the test compounds as described above. To measure the cytokine level after 24 h of cultivation, the culture medium was harvested, and the cell monolayer was lysed. Then the supernatant and lysates were combined and the content of some proinflammatory interleukins was determined using specific test kits in accordance with the manufacturer’s protocol. The level of pro-IL-1β was determined by immune blotting techniques.

LPS predictably causes an increase in the production of the proinflammatory cytokine TNF in mouse macrophages, which indicates the induction of an inflammatory process in these cells. Incubation of macrophage cells with both 1,4-NQs in the presence of LPS led to significant suppression of TNF secretion. Compound **U-573** was the most effective inhibitor of TNF production, and at concentrations of 0.1–1.0 μM, it inhibited the production of this cytokine by 60–80% compared with control cells incubated with LPS alone. Compound **U-443** at both tested concentrations inhibited TNF production by 40–45% (Figure 2E). In both cases, studied compounds exhibited full protection (*p* < 0.05 compared with control intact cells), while **U-573** at a concentration of 0.1 μM significantly reduced TNF production even below the control value of intact cells.

The level of pro-IL-1β in macrophages in the presence of LPS was also significantly reduced by the two 1,4-NQs as shown in Figure 2F. Compound **U-443** and **U-573** at 1.0 μM downregulated the pro-IL-1β expression by 18% and 80% relative to control cells exposed to LPS alone. 

#### 2.3.3. Determination of 1,4-NQs Antioxidant Activity in Brain Homogenate

**Figure 3:**
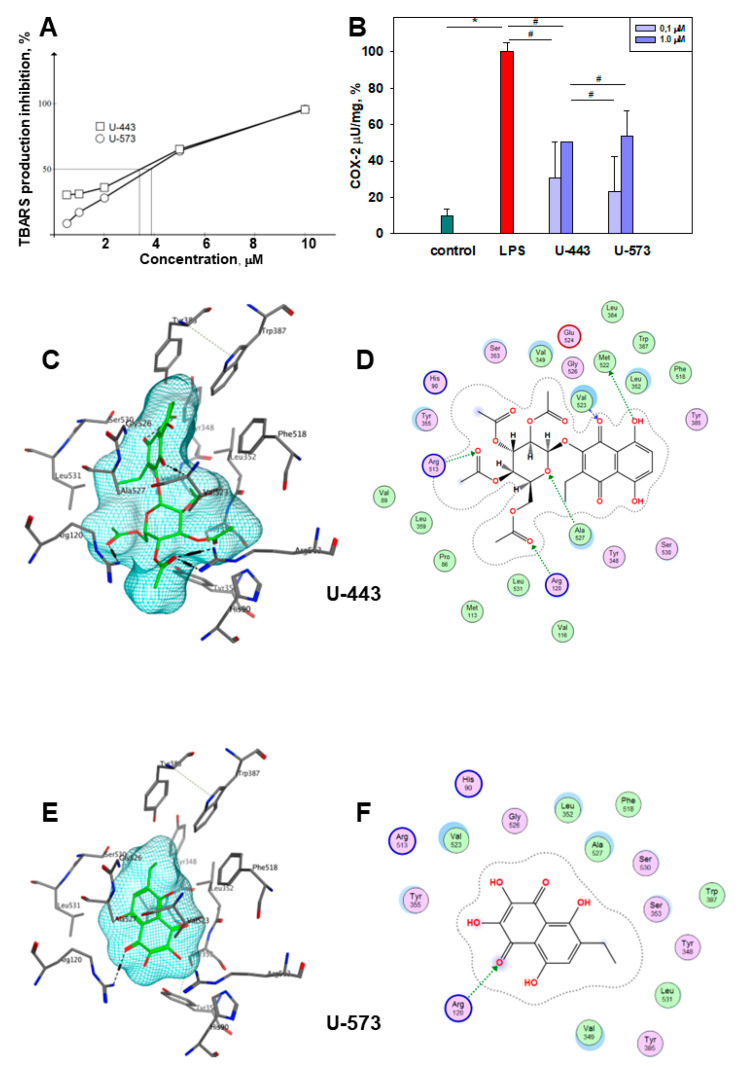
The impact of **U-443** and **U-573** on the iron-induced oxidation of the mouse brain homogenate (**A**) and the activity of mouse COX-2 (**B**). The color codes within the figure indicate the concentrations of the tested compounds in µM. The data are presented as mean ± SEM (n = 3). * *p* < 0.05 indicates a significant difference compared with control cells, while ^#^ *p* < 0.05 indicates a significant difference compared with cells treated with LPS alone. Included in the figure are the binding diagrams and 2D contact diagrams between **U-443** (**C**,**D**) and **U-573** (**E**,**F**) with the mCOX-2 active site (PDB ID 1CX2). The ligands are depicted in the green stick form, while the molecular surface of the ligands is shown as blue lines.

The antioxidant properties of the synthetic 1,4-NQs were investigated using a model involving the nonenzymatic Fe^2+^-induced oxidation of mouse brain homogenate (Figure 3A). To evaluate antioxidant activity, a fluorescent method was employed to measure thiobarbituric acid reactive substances (TBARS), which are the end products of oxidation that react with thiobarbituric acid and are formed due to the involvement of various reactive oxygen species. Preincubation of mouse brain homogenate with the selected compounds, **U-443** and **U-573**, resulted in a decrease in TBARS content following Fe^2+^-induced oxidation. Compound **U-443** exhibited a dose-dependent reduction in TBARS levels, ranging from 30.3% to 95.6% at concentrations of 0.05–10.0 µM, with an EC_50_ value of 3.4 µM. Similarly, compound **U-573** demonstrated a nearly linear dose-dependent efficacy across the entire concentration range of 0.05–10.0 µM, leading to a reduction in TBARS content from 8.6% to 95.9%, with an EC_50_ value of 3.9 µM. The antioxidant activity of the tested 1,4-NQs was slightly lower than that of ionol, chosen as the reference antioxidant substance, which exhibited approximately 90% inhibition of TBARS content at a concentration of 0.5 µM (data not shown, EC_50_ = 0.27 µM).

### 2.4. Influence of 1,4-NQs on COX-2 Activity

According to the obtained data, LPS activated cyclooxygenase-2 (COX-2) in RAW 264.7 cells. This is consistent with the hypothesis that this enzyme, the expression of which is found in macrophages, is inducible and it begins to function actively during inflammation after exposure to lipopolysaccharides, cytokines, and growth factors. We found that both 1,4-NQs at concentrations of 0.1–1.0 μM were capable of significantly inhibiting COX-2 activity in LPS-preactivated macrophages. The most active compound was **U-573**, which at a concentration of 0.1 μM reduced the activity of this enzyme by approximately 80% compared with LPS-stimulated cells (Figure 3B). No significant dose-dependence effect was found for both studied compounds—although, at a concentration of 0.1 μM, they were insignificantly more active. At a concentration of 0.1 μM, both compounds almost completely inhibited COX-2 activity (*p* < 0.05 compared with enzyme activity in the control).

Molecular docking with the active site of cyclooxygenase was performed for 1,4-NQs **U-443** and **U-574**, which inhibit mCOX-2. Mouse cyclooxygenase-2 was chosen for 1,4-NQ molecular docking because we tested enzyme activity in mouse RAW 264.7 macrophage cells and carried out further studies in vivo in mouse models of Parkinson’s disease. It was found that the **U-573** compound, when interacting with the active site, forms a hydrogen bond with Arg 120 (Figure 3E,F). Compound **U-573** is small in comparison with the size of the catalytic center and its localization near Arg 120 could explain the inhibitory effect of **U-573** on mCOX-2. Molecular docking of the **U-443** compound with mCOX-2 using the SC-558 selective inhibitor binding site as a template showed that the **U-443** carbohydrate fragment, when interacting with the active site, forms hydrogen bonds with Arg 120, Arg 513, and Ala 527, and the naphthoquinone fragment interacts with residues Met 522 and Val 523 (Figure 3C,D). The binding affinity of compounds **U-573** and **U-443** to COX-2 is −7.83 and −12.31 kcal/mol, respectively. This confirms the binding of the studied naphthoquinones to the active center of cyclooxygenase-2.

### 2.5. Behaviour Assessments of Spontaneous Locomotion in An In Vivo Murine Rotenone-Induced Parkinson’s Disease Model

#### 2.5.1. Acute Toxicity of The Studied 1,4-NQs Estimation

The acute toxicity of the drugs was assessed using the Karber formula, calculating doses that cause the death of 50% and 100% of animals (LD_50_ and LD_100_ respectively). The studied 1,4-naphthoquinones were injected into CD1 mice in olive oil once intraperitoneally. The animals were observed for 14 days. Indicators of acute toxicity for the tested compounds are as follows:

**U-443**, LD_100_ > 100 mg/kg;
**U-573**, LD_100_ > 100 mg/kg.

**Figure 4:**
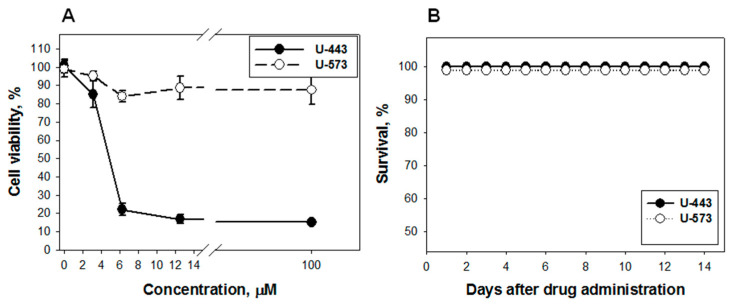
Cytotoxic effect of **U-443** and **U-573** on the viability of Neuro-2a cells (**A**). Adhered Neuro-2a cells (1 × 10^4^ cells/well) were incubated in a CO_2_ incubator at 37 °C for 24 h. Subsequently, different concentrations of 1.4-NQs solution (20 µL) were added to the cells and incubated for an additional 24 h. Cell viability was then assessed using the MTT assay. The data are presented as mean ± SEM (n = 3). Mouse survival (%) after drug administration is shown in (**B**). Intraperitoneal injections of 1.4-NQs solutions were administered to the mice at a maximum inspected dose of 100 mg/kg in a 0.1 mL volume. The control and experimental groups, each consisting of 6 mice, were observed for a period of 14 days.

Thus, compounds **U-443** and **U-573** were found to be of low toxicity since they did not show any toxic properties at a dose of 100 mg/kg (Figure 4B).

#### 2.5.2. Animal Spontaneous Locomotion Assessments

**Figure 5:**
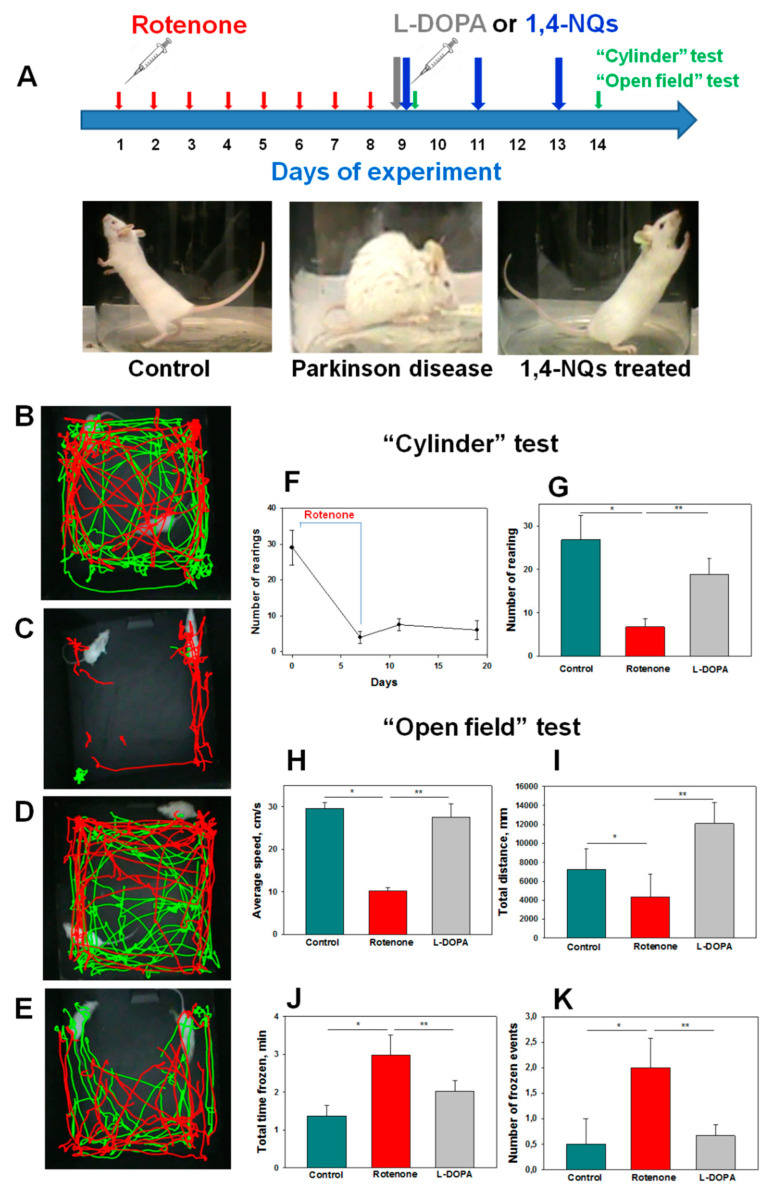
Influence of levodopa (L-DOPA) and 1,4-NQs on spontaneous locomotion in mice with rotenone-induced Parkinson’s disease. Scheme of experiments (**A**). Movement path of two untreated mice (**B**), after rotenone treatment (6 mg/kg, 8 days, **C**), after rotenone and L-DOPA (5 mg/kg) treatment (**D**), and after rotenone and **U-573** (1 mg/kg) treatment (**E**) in “Open field” test. Effect of rotenone treatment on mouse behavior in “Cylinder” test during 19 days (**F**). Impact of L-DOPA on the number of rearings after rotenone treatment in the “Cylinder” test (**G**) and on mouse average speed (**H**), total distance (**I**), total time motionless (**J**), and freezing events (**K**) in the “Open field” test. Data are presented as mean ± sd (n = 6); * *p* < 0.05 compared with the control group of mice; ** *p* < 0.05 compared with the group of mice treated with rotenone.

In the first stage of the research, a rotenone-induced experimental in vivo model of the early stage of parkinsonism was created. For this purpose, a solution of rotenone in olive oil was injected into CD1 mice at a dose of 6 mg/kg subcutaneously once a day for 8 days (Figure 5A). Then, the study drugs were administered. A solution of levodopa (L-DOPA) used as a reference drug in saline at a dose of 5 mg/kg was administered orally once a day after the last dose of rotenone. Then, after 40 min, the experiment was evaluated to compare the effect with L-DOPA. The behavior of the animals was assessed in the “Cylinder” (mouse investigation of the horizontal and vertical surfaces of the cylinder wall) and “Open field” (a study of mouse motor and orientation-exploratory activity) tests. The number of mice getting up on their hind legs in the “Cylinder” test was recorded on a video camera and subsequently counted visually. To register (tracking) behavior in the “Open field” test, we used ToxTrac software, v.2.84.

As shown in Figure 5, rotenone significantly and reliably altered almost all indicators of animal behavior. Namely, it reduced the number of rearings, the speed of movement, and the total distance traveled, and significantly increased the time without movement (total time frozen) and the number of freezing events by the mice. The suppressive effect of rotenone, assessed in the “Cylinder” test, remained virtually unchanged for 10 days after the last dose of rotenone administration (Figure 5F).

L-DOPA significantly and reliably returned the noted parameters of the animal’s neurological status to the control level, thereby providing neuroprotective (antiparkinsonian) effects. This drug significantly increased the number of rearings in the “Cylinder” test, the average speed and total distance parameters, and markedly reduced the number of motionless events and the total time spent frozen in the “Open field” test. The differences between L-DOPA and the control group were not statistically significant.

In subsequent experiments, we investigated the antiparkinsonian activity of 1,4-NQs, **U-443** and **U-573**, in a rotenone-induced experimental in vivo model of PD in mice. For this purpose, PD induction with rotenone was performed as described above. Compounds **U-443** and **U-573** in doses of 0.1, 1.0, or 10.0 mg/kg were administered starting one day after the last injection of rotenone, intraperitoneally, three times every other day. Evaluation of the effectiveness was carried out one day after the last injection of 1,4-naphthoquinones in the tests “Cylinder” and “Open field”.

**Figure 6:**
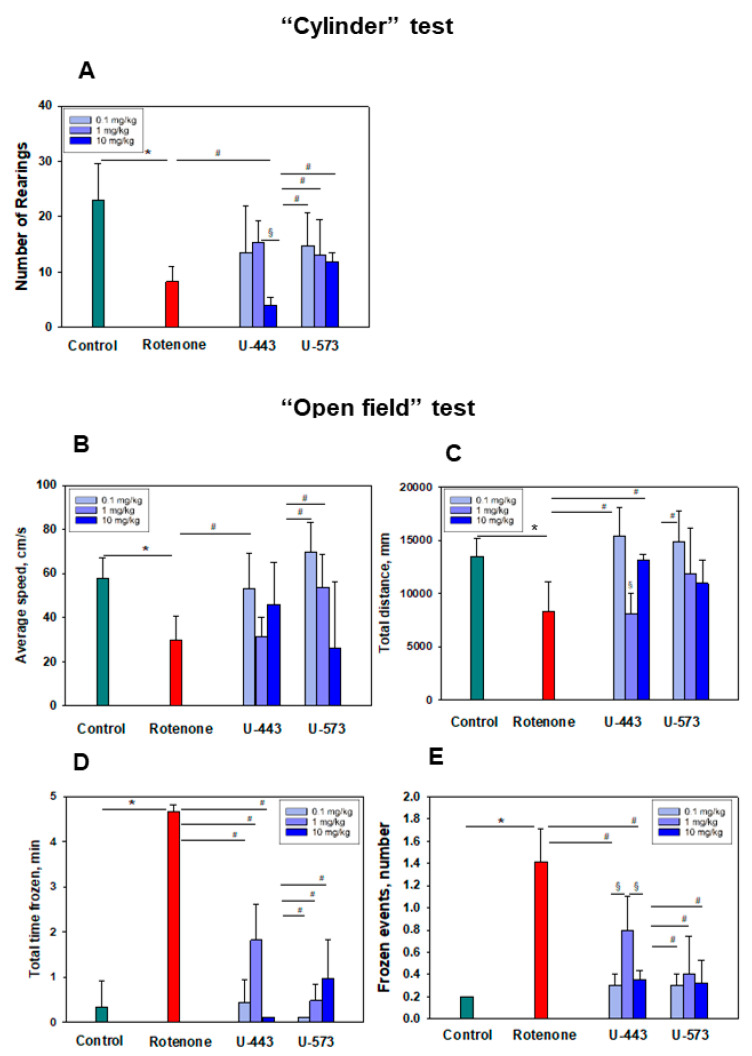
Influence of 1,4-NQs on spontaneous locomotion in mice with rotenone-induced Parkinson’s disease. Effect of 1,4-NQs **U-443** and **U-573** on the number of mouse rearings after rotenone treatment in the “Cylinder” test (**A**), on the mouse average speed (**B**), total distance (**C**), total time frozen (**D**) and frozen events (**E**) in the “Open field” test. Data are presented as mean ± sd (n = 6); * *p* < 0.05 compared with the mouse control group; ^#^ *p* < 0.05 compared with the mouse group treated with rotenone alone; ^§^ *p* < 0.05 compared between mouse groups treated with different doses of each compound.

In the “Cylinder” and “Open Field” tests, rotenone significantly (*p* < 0.05) reduced the number of rearing of mice, average speed, and total distance, and increased the total time frozen and frozen events number (Figure 6A–E). Compound **U-443** at doses of 0.1 and 1.0 mg/kg had no significant effect on the number of rearing compared with rotenone. No statistical difference in effect was found between these doses. But at a dose of 10 mg/kg, this naphthoquinone significantly reduced this parameter compared with rotenone and compared with the other two doses. At the same time, the compound **U-573** significantly improved the number of rearing in comparison with rotenone at all studied doses of 0.1–10.0 mg/kg. However, no statistical difference in effect between different doses of **U-573** was found. (Figure 6A).

In the “Open Field” test, compound **U-443** significantly (*p* < 0.05) improved average speed (0.1 mg/kg), total distance (0.1 and 10.0 mg/kg), total time frozen (all doses), and the number of frozen events (0.1 and 10.0 mg/kg) (Figure 6B–E). A significant difference between **U-443** doses was found between 1.0 mg/kg and the other two doses when the total distance and the number of frozen events were determined (Figure 6C,E). In the case of **U-573,** this compound, in fact, at all doses used (except 10 mg/kg in the average speed measurement and 0.1 and 10.0 mg/kg in the total distance) significantly improved all recorded parameters in comparison with rotenone in the “Open Field” test. However, no statistical difference in the effects was found between all doses (0.1–10.0 mg/kg) used in the experiment. 

When comparing the results of the action of **U-573** on animals with induced parkinsonism with control, it was found that the effects of used doses of 0.1 and 1.0 mg/kg (number of rearing and average speed), 0.1 mg/kg (total distance), 0.1–10.0 mg/kg (total time frozen) and 0.1–10.0 mg/kg (the number of frozen events) did not statistically differ from those of intact animals but were significantly different from rotenone-treated mice. In the case of **U-443,** the effects of doses of 0.1 mg/kg (number of rearing, average speed), 0.1 and 10.0 mg/kg (total distance), and 0.1–10.0 mg/kg (total time frozen and the number of frozen events ) did not significantly differ from intact animals but differed significantly from rotenone-treated mice. This may indicate that studied compounds at low doses contribute to the considerable recovery of cognitive functions and behavioral responses in animals injected with rotenone to almost baseline values without rotenone. 

## 3. Discussion

In this work, we selected two 1,4-naphthoquinones, synthesized sea urchin pigment **U-573** and synthetic acetylglucoside **U-443** of ophiuroid naphthazarine pigment **6**, which are the most effective in a preliminary screening of cytoprotective activity in vitro model of neurotoxicity induced by rotenone. Ethylspinazarin **U-573** is a 6-deoxy analog of 6-ethyl-2,3,5,7,8-pentahydroxy-1,4-naphthoquinone **U-138** (echinochrome from sea urchin *Scaphechinus mirabilis*) multifaceted medicine [28]. Ethylspinazarin and echinochrome are structurally related quinones and both have excellent antioxidant properties [28,29]. Echinochrome **U-138** and ethylspinazarin**U-573** have two acidic β–hydroxyl groups at the 2 and 3 positions of the ethylene bond conjugated with quinone carbonyl [30].

Unlike ethylspinazarin **U-573**, the structure of the acetyl-*O*-glucoside derivative **U-443** does not contain free β-hydroxyl groups. The compound **U-443**, a glucoside derivative, is a simplified analog of the bioactive echinochrome acetylated tris-*O*-glucoside **U-133**. Previous research [31] has demonstrated the therapeutic potential of **U-133** in preventing and/or slowing down neurodegeneration similar to Parkinson’s disease. **U-443** serves as a more readily available alternative with similar therapeutic properties.

A number of synthesized 1,4-naphthoquinones, including **U-573,** have already demonstrated pronounced neuroprotective properties in paraquat- and 6-OHDA-induced models of Parkinson’s disease in vitro [22]. In the present work, it was found that the synthetic 1,4-naphthoquinones, **U-443** and **U-573**, are also capable of partially protecting neuronal cells from the cytotoxic effect of the neurotoxin rotenone. These 1,4-NQs have a modest impact on the level of some oxidative burst products and proinflammatory agents under rotenone and LPS influence. The fact that the studied 1,4-naphthoquinones similarly protect neuronal and macrophage cells from the damaging effects of both rotenone and LPS may indicate the similar molecular mechanisms of the protective action. Most likely, the inhibition of ROS production in these cells under rotenone and LPS actions is the key step that prevents the development of additional events such as MMP depolarization, mitochondrial dysfunction, proinflammatory cytokine release, DNA damage, apoptosis induction, and some other perturbations leading to cell death. Compounds **U-443** and **U-573** were used at concentrations less than EC_50_ values, which did not suppress cell viability. These 1,4-NQs by themselves did not significantly affect the levels of ROS and NO in cell cultures, mitochondrial membrane potential, TNF production, and COX-2 activity (data not shown).

Evidently, **U-443** and **U-573** have the ability to hinder the production of reactive oxygen species in neuronal cells or macrophages, thereby regulating the activity of enzymes responsible for maintaining a balanced level of reactive oxygen species within cells. These compounds can stimulate various antioxidant enzymes, such as superoxide dismutase, catalase, and peroxiredoxins, to eliminate excessive reactive oxygen species. Additionally, they inhibit enzymes involved in the generation of ROS, including NADPH oxidase in phagocytic cells, xanthine oxidase, mitochondrial cytochrome C oxidase, and microsomal monooxygenases. These effects can be attributed to the activation of the Keap1/Nrf2/ARE signaling pathway by 1,4-naphthoquinones, which help regulate the cellular redox status [18,22]. At the same time, we showed that **U-443** and **U-573** partially block NO formation in RAW 264.7 cells. Perhaps this is due to the targeted inhibition of inducible iNO synthase, which is responsible for the synthesis of nitrogen monoxide in macrophages after stimulation.

In recent studies, it has been demonstrated that several 1,4-naphthoquinone derivatives synthesized by us effectively inhibit the activity of purinergic P2X7 receptors (P2X7R) in vitro. The inhibition results in marked blockade of the receptor macropore, a significant reduction in P2X7R-mediated production of ROS, NO, release of NLRP3 inflammasome-dependent proinflammatory cytokines, and pronounced protection of neuronal and macrophage cells from the ATP toxic impact. For one of the 1,4-NQ derivatives, compound **U-556**, direct binding to this purinoceptor has been proven by surface plasmon resonance. In silico analysis showed the ability of some synthetic 1,4-NQs to specifically bind to an allosteric site located in the extracellular region of P2X7R and to almost completely eliminate receptor-mediated inflammation in mice [23,32,33]. Thus, inhibition of P2X7R activity may also contribute to the compounds **U-443** and **U-573** protective potential.

Several natural and synthetic 1,4-naphthoquinones have been documented to possess notable antioxidant activity [15]. In the present study, compounds **U-443** and **U-537** demonstrated a pronounced inhibition of the induced peroxide radical production in mouse brain homogenate. Moreover, this capability may contribute to the reduction of reactive oxygen species formation and the attenuation of neuroinflammation in mouse brain tissue, which are commonly observed in the initiation and advancement of Parkinson’s disease.

In the investigated concentration range, it was noted that the cytoprotective effects of **U-443** and **U-537** compounds do not depend on the dosage. It is now understood that many quinones display dual effects on biological systems and demonstrate nonlinear, U-shaped dose–response curves. The action of quinones, dependent on interaction duration and dose, can result in either toxicity or cytoprotection. At lower doses, quinones with cytoprotective properties activate detoxification enzymes through electrophilic responses. Conversely, at higher doses, less selective and more reactive quinones exhibit cytotoxic properties by disrupting cell functions through the generation of semiquinone radicals and superoxide, ultimately leading to the formation of highly toxic hydroxyl radicals causing cell death [34].

We found that, in some cases, **U-443** at the highest studied concentration of 1 μM exhibited the opposite cytoprotective effect. This concentration is close to its cytotoxicity EC_50_ value. Upon closer inspection, this impacts the manifestation of its cytotoxic activity (Figure 1B) and results in slight hyperpolarization, indicating the change in MMP (Figure 1E) and increase in ROS and NO production (Figure 2A,B) at this concentration when co-cultivated with the neurotoxin rotenone. This may be explained by the weak cytotoxic impact of compound **U-443** since a concentration of 1 μM is quite near to the onset of its cytotoxic activity. 

Currently, it is known that some 1,4-NQs can exhibit cytotoxic properties due to the induction of semiquinone radicals and superoxide formation in exposed cells followed by affected redox signaling, alkylation, DNA damage, cell cycle blockade, and subsequent apoptosis. Some naphthoquinones have been shown to be cytotoxic to neuronal cells. Thus, natural 8-hydroxy-2-methoxy-1,4-naphthoquinone and 5-hydroxy-2-methoxy-1,4- naphthoquinone from plant *Juglans sinensis* and series of synthetic 1,4-NQs derivatives exhibited significant cytotoxicity against human SH-SY5Y and mouse Neuro-2a neuroblastoma cells [21,35]. Exposure to some 1,2-NQs и 1,4-NQs provokes human neuroblastoma cell lines SK-N-SH apoptosis in a ROS-dependent pathway, including DNA damage, mitochondrial dysfunction, and resultant caspase 3 and 9 activation together with GSH decrease, proteasome inactivation and NQO1 upregulation [36]. Treatment of mouse hippocampal neuronal HT22 cells with 2,3-dimethoxy-1,4-naphthoquinone increased cytosolic and mitochondrial ROS and apoptosis, involving CHOP/GADD153 upregulation, JNK and p38 MAPK activation, Bak/Bax activation, mitochondrial membrane potential loss, caspase-9 and caspase-3 activation, PARP cleavage, nucleosomal DNA fragmentation, and intracellular GSH reduction [37].

In our previous investigations, we showed that the antiproliferative and cytotoxic activity of a series of 1,4-NQ derivatives against mouse neuronal cells largely related to their hydrophobicity/polarity, depending among other things on the acetylation of the sugar moiety of 1,4-NQ glycosides [21]. The observed difference in cytotoxic activity between compounds **U-443** and **U-537** can be explained by differences in the nature of the substituents of the naphthoquinone core. Thus, unlike polyhydroxy-1,4-NQ **U-537**, compound **U-443** contains a bulky lipophilic acetylglycoside fragment. Previously, we showed [21] that the introduction of an acetylglycoside fragment leads to a cytotoxic effect increasing in comparison with the initial hydroxyquinone. Deacetylation of such glycosides leads to a several-fold cytotoxicity decrease. 

Many quinones exhibit ambivalent and opposite effects on biological systems. Depending on the dose used, cellular targets, and exposure time, the effect of quinones on cells leads to toxicity or cytoprotection. In contrast to the cytotoxic activity of naphthoquinones at high concentrations, at low doses, stable and selective quinones often exhibit cytoprotective properties resulting from electrophilic counterattacks, leading to the detoxification enzymes’ appearance through the Keap1/Nrf2/ARE pathway, and scavenging action [34,38].

It has been established that a number of natural and synthetic naphthoquinones, including 1,4-naphthoquinone derivatives, are able to protect various types of cells from adverse effects. For example, 2-carbomethoxy-2,3-epoxy-3-prenyl-1,4-naphthoquinone (CMEP-NQ) has been found to effectively save human acute leukemia Jurkat T cells from some apoptotic cell death inducers, such as microtubule-damaging and DNA-damaging agents. The cytoprotection of CMEP-NQ was provided by the elevation of anti-apoptotic chaperone regulator BAG3 and cell differentiation protein MCL-1 levels, which leads to the mitochondrial apoptosis pathway inhibition, as well as by blocking mitochondrial damage caused by intracellular ROS production [39]. Several novel short-chain 2,3-disubstituted naphthoquinone derivatives were found to protect human hepatic carcinoma HepG2 cells and rodent retinal precursor RGC5 cells against loss of viability and cell death in the presence of rotenone by protecting and restoring mitochondrial dysfunction caused by this toxin [40]. Echinochrome (**U-138**) was described recently to protect the primary culture of rat pulmonary fibroblasts from oxidative stress caused by hydrogen peroxide H_2_O_2_, probably due to the increase in mitochondrial biogenesis, rise of chaperone Hsp70 activity, and stimulation of hypoxia-inducible factor HIF-1 expression, which together significantly enhance the resistance of cells to damage [41]. Ubiquinone short-chain synthetic analog, idebenone, was also shown to effectively protect human retinal pigment epithelia ARPE-19 cells exposed to H_2_O_2_ inducing oxidative damage. Idebenone cytoprotective action was accompanied by Keap1/Nrf2/ARE signaling pathway activation, reduction of the Bax/Bcl-2 ratio, recovery of the mitochondrial membrane potential to physiological levels, preservation of the ROS production, and minimization of caspase-3 activity [42]. Plumbagin obtained from the roots of different medicinal plants was demonstrated to effectively protect neuronal cells and chondrocytes exposed to H_2_O_2_, which was accompanied by an enhancement in antioxidant enzyme activities and the expression of Nrf2; it also prevents dexamethasone-induced apoptosis in osteoblasts by restoring mitochondrial potential and has a pronounced neuroprotective effect on neuronal human SH-SY5Y cells, hepatoprotective potential leading to liver regeneration, as well as cardioprotective activity on rat H9c2 cardiomyocytes. In addition, the powerful anti-inflammatory activity of plumbagin has been studied in sufficient detail. According to numerous investigations performed on various immune cells, including murine lymphocytes, RAW264.7 macrophages, bone marrow-derived macrophages, microglia, and some others, this natural naphthoquinone exhibited its protective anti-inflammatory properties by normalizing an imbalance of ROS/NO, antioxidant molecules CAT, GPx and SOD, and proinflammatory cytokines TNF, IL-1β, IL-6, and IL-12, as well as an increase in Nf-kB oxidation. Raised expression of iNOS, granulocyte-colony stimulating factors G-CSF, monocyte chemoattractant protein MCP1, and MCP5 was also reverted by plumbagin treatment [43]. Thus, protective properties are inherent in many naphthoquinones, which they exhibit on various types of cells.

The moderate inhibition of proinflammatory cytokine TNF and pro-IL-1β production by 1,4-naphthoquinone derivatives, following exposure to LPS found in the present study, may take place either at the level of the TLR4 receptor or in the downstream signaling pathway. Recently, it has been discovered that 1,4-naphthoquinones effectively block the interleukin-1 receptor-associated kinase (IRAK1) in LPS-stimulated human THP-1 macrophages. IRAK1 kinase plays a crucial role as a signaling intermediate in the IL1R/TLR downstream cascade, activating various transcription factors that contribute to the regulation of immune responses and inflammation. The interaction between certain 1,4-naphthoquinones and IRAK1 has been shown to inhibit the production of proinflammatory cytokines such as TNF, IL-1β, IL-6, IL-8, and IL-10 in macrophages [44]. In our experiments, the minimal presence of IL-1β detected following cell stimulation with LPS is associated with the particular behavior of RAW 264.7 cells. These macrophages are known to release pro-IL-1β but not mature IL-1β due to the absence of the apoptotic speck-like protein required for the further processing of pro-IL-1β into its mature form [45].

LPS-induced COX-2 activation can be inhibited in several ways. First, effective 1,4-NQs can directly interact with cyclooxygenase-2 and selectively inhibit the activity of the enzyme due to the binding of the catalytic center responsible for the activity of cyclooxygenase to amino acids, especially Arg 120, similar to the known inhibitor of this enzyme, SC-558 [46]. Binding to Arg 120 plays a pivot role in the interaction of COX-2 with the substrate and with inhibitors [47,48]. Such activity has been previously noted for a number of 1,4-naphthoquinone derivatives [49,50,51]. Our data on modeling (docking) of compounds **U-443** and **U-573** clearly indicate the direct interaction of these 1,4-NQs with the active center of COX-2, which may be the reason for the manifestation of their properties inhibiting enzymatic activity. Second, suppression of the production of a number of cytokines noted in our study can also directly affect COX-2 and be a direct cause of a decrease in COX-2 activity.

Rotenone with high efficiency blocks the transfer of electrons in complex I of the mitochondrial chain. Under the action of rotenone throughout the brain, a general suppression of Complex I occurred, as a result of which, in the group of animals receiving rotenone, signs of PD appeared, such as the motor deficit, selective nigrostriatal dopaminergic degeneration, and formation of ubiquitin- and synuclein-positive nigral inclusions. The toxic effect on cells caused by rotenone-induced oxidative stress can initiate neurodegeneration processes in Parkinson’s disease [52]. The process of mitochondrial respiration and dopamine metabolism leads to the formation of reactive oxygen species. In Complex I, near the rotenone binding site, there is a site through which an electron leakage passes, which promotes the formation of ROS; disruption of the functioning of Complex I leads to an increase in ROS [53]. In addition to oxidative stress in neurons, rotenone promotes activation of the microglia surrounding neurons. In the early and progressive stages of PD, astrocytes and microglia are activated in various ways. This, in turn, contributes to an increase in the number of dead neurons, which leads to an increase in the level of superoxide anions, free radicals, IL-1β, TNF, PGE2, NO, and other substances. The combination of these processes initiates dopaminergic neurodegeneration [54].

Both tested compounds showed comparable efficacy as antiparkinsonian agents in the “Cylinder” and “Open Field” tests. It is highly probable that during the early stage of Parkinson’s disease induction by rotenone in live organisms, the investigated compounds **U-443** and **U-573** exhibit antiparkinsonian properties through their capability to decrease the presence of reactive oxygen species and NO in neurons and microglia. These compounds also demonstrate the ability to inhibit the production of proinflammatory cytokines and suppress the activity of COX-2, resulting in cytoprotection, enhanced neuronal survival, and reduced inflammatory processes in microglia.

We did not find a clear dose dependence of the antiparkinsonian activity of 1,4-naphthoquinones or their effects on the behavioral responses of animals with induced PD in the dose range of 0.1–10.0 mg/kg. Despite the fact that the studied naphthoquinones do not exhibit acute toxic properties in animals up to a dose of 100 mg/kg, the use of drugs at lower doses of 0.1–1.0 mg/kg seems to be preferable in future experiments.

In a previous study employing QSAR methodologies, it was observed that the neuroprotective activity of the investigated compounds relies on the hydrophobicity, polarity, charge, and shape of the 1,4-naphthoquinone molecule. These aforementioned properties suggest that these small molecules have the potential to effectively penetrate the blood–brain barrier and reach neurons and microglial cells, consequently offering neuroprotection in animal models of Parkinson’s disease induced by rotenone.

Detailed studies of the cytotoxicity of these 1,4-NQs in relation to Neuro-2a cells were carried out and published by us earlier [21]. For compound **U-573,** cytotoxicity was established (EC_50_ > 100 µM), which indicates a very weak cytotoxic effect, while **U-443** was more cytotoxic (EC_50_ = 4.46 µM), which excludes this substance from candidates for promising cytoprotectors. At the same time, compound **U-573** exhibits cytoprotective properties against Neuro-2a cells in the presence of various neurotoxins, such as paraquat, 6-OHDA, or rotenone, at concentrations much lower than cytotoxic ones, 0.1 and 0.01 µM [22]. Compound **U-573** has very low toxicity in animals (LD_100_ > 100 mg/kg), and in an in vivo study, in many cases, this compound at low doses showed statistically significant effects. This allows us to consider 1,4-naphthoquinone **U-573** as a promising small biomolecule for further investigation of its neuroprotective properties and ability to prevent and correct neurodegenerative disorders.

## 4. Materials and Methods

### 4.1. Synthesis of 1,4-Naphthoquinones General

6-Ethyl-2,3-dihydroxynaphthazarin **4** (**U-573**) was synthesized from ethyldichloronaphtazarin as described in our works [22,24]; 2-Hydroxy-3-ethylnaphthazarin **6** was obtained by reductive dechlorination of appropriate dichlorohydroxyethylnaphthazarin **5** according to our previous paper [26]; the synthesis of 3,4,6-tri-*O*-acetyl-α-d- glucopyranose 1,2-(tert-butoxy orthoacetate) **7** was accomplished using Kochetkov’s method, as described in reference [26]; 2-Hydroxy-3-ethylnaphthazarin **6** was condensed with 3,4,6-tri-*O*-acetyl-α-d-glucopyranose 1,2-(tert-butoxyorthoacetate) **7** in chlorobenzene at reflux and led to acetylglucoside **8** (**U-443**) as described in our previous works [17,25]. NMR spectra for **4** (**U-573**) were recorded on a Bruker AVANCE-500 at frequencies 500 MHz for ^1^H spectra and 125 MHz for ^13^C in DMSO-d_6_. NMR spectra for **8** (**U-443**) were recorded on a Bruker AVANCE-700 at frequencies 700 MHz for ^1^H spectra and 176 MHz for ^13^C in CDCl_3_ (Figures S1 and S2 in Supplementary Materials). The purity of the synthesized compounds was assessed on LaChrom (Merck Hitachi, Tokyo, Japan) system (pump L-7100, UV/VIS detector L-7400, column oven L-7300, and integrator D-7500). Separations were carried out using Agilent Technologies column Zorbax Eclipse XDB-C18, 5 μm (150 mm × 4.6 mm) with a guard column Hypersil ODS (4.0 mm × 4 mm). The binary gradient employed phase A: water/glacial acetic acid (v/v, 100/1); phase B: acetonitrile/glacial acetic acid (v/v, 100/1) according to the following profile: 0–35 min, 5–35% B. The flow rate was 1 mL/min. The operating temperature was set at 30 °C, and the detection wavelength was set at 480 nm (Figures S3 and S4).

1,4-NQs were prepared as a 10 mM stock solution in DMSO and then dissolved in ddH_2_O to the final concentrations before experiments.

### 4.2. Cell and Cultivation Conditions

Neuro-2a CCL-131^TM^ mouse neuroblastoma cell line and RAW 264.7 TIB-71™ mouse macrophage cell line were obtained from ATCC (American Type Culture Collection, Manassas, VA, USA). The cells were cultured in DMEM medium (Biolot, St. Petersburg, Russia), which was supplemented with 10% fetal bovine serum (Biolot, St. Petersburg, Russia) and 1% penicillin/streptomycin (Biolot, St. Petersburg, Russia). The cultures were maintained in a CO_2_ incubator at a temperature of 37 °C with 5% CO_2_.

### 4.3. Cell Viability Assessment (MTS Test)

Neuro-2a or RAW 264.7 cells (1.0 × 10^4^/200 µL) were seeded into 96-well plates and cultured at 37 °C in a 5% CO_2_ incubator for 24 h. Afterward, **U-443** or **U-573** compounds were added to the cell wells at concentrations of 0.01, 0.1, and 1.0 μM. The plates were then incubated for 1 h. Then, a solution of rotenone (Sigma-Aldrich, St. Louis, MO, USA) at a final concentration of 10.0 μM was added to the wells. This concentration of rotenone suppressed cell viability by around 30–40%. After 24 h cytoprotective effect of compounds was determined by the MTS assay. The absorbance in each well was detected at 490/630 nm using a PHERAstar FS plate reader (BMG Labtech, Ortenberg, Germany). The experiments were conducted in triplicate and the results were expressed as the percentage of inhibition relative to the untreated cells exposed to rotenone.

To study the cytotoxic activity of **U-443** or **U-573**, these compounds were added at final concentrations of 0.01–100.0 μM to the wells of 96-well plates with Neuro-2a cells and the cells were incubated for an additional 24 h. Cell viability was then determined as described above.

### 4.4. Measurement of Mitochondrial Membrane Potential (MMP)

Neuro-2a cells (1.0 × 10^4^ cells/well in a 96-well plate) were treated with different concentrations of compounds **U-443** or **U-573** (0.01–1.0 μM) for 1 h at 37 °C in a 5% CO_2_ incubator. Following this, a solution of rotenone at a concentration of 10.0 μM was added, and the cells were further incubated for 1 h. Subsequently, a solution of tetramethylrhodamine methyl (TMRM) at a concentration of 500 nM (Sigma-Aldrich, St. Louis, MO, USA) was added to each well and incubated for 30 min at 37 °C. Fluorescence intensity was measured using a PHERAstar FS plate reader (BMG Labtech, Ortenberg, Germany) with excitation at 540 nm and emission at 590 nm. Data analysis was performed using MARS Data Analysis v. 3.01R2 (BMG Labtech, Ortenberg, Germany), and the results were presented as a percentage of the control [55].

### 4.5. Analysis of ROS and NO Levels

Cells (1.0 × 10^4^ cells/well) were treated with compounds **U-443** or **U-573** at concentrations ranging from 0.01 to 1.0 μM for 1 h. Subsequently, rotenone was added to each well at a final concentration of 10.0 μM, and the cells were incubated for an additional 1 h. To evaluate the production of ROS, a 20 μL solution of 2,7-dichlorodihydrofluorescein diacetate (H2DCF-DA, Molecular Probes, Eugene, OR, USA) at a final concentration of 10.0 μM was added to each well, and the plates were further incubated for 10 min at 37 °C in the absence of light.

To evaluate the nitric oxide levels in cells, a solution of the NO-sensitive probe DAF-FM (Molecular Probes, Eugene, OR, USA) was introduced to each well at a concentration of 5.0 μM. The plates were then incubated for 40 min at 37 °C in the absence of light. Fluorescence intensity was measured using a PHERAstar FS high-speed plate reader (BMG Labtech, Ortenberg, Germany) with excitation at 485 nm and emission at 518 nm. The obtained data were analyzed using MARS Data Analysis v. 3.01R2 (BMG Labtech, Ortenberg, Germany), and the results were expressed as a percentage of the control [55]. 

### 4.6. TNF ELISA Assay 

RAW 264.7 cells (2.0 × 10^4^/200 µL) were plaсed in 96-well plates and allowed to adhere for 2 h at 37 °C in a 5% CO_2_ incubator. Following the adhesion period, cells were treated with compounds **U-443** or **U-573** at concentrations ranging from 0.1 to 1.0 µM for 1 h. Subsequently, LPS (*Escherichia coli* 055:B5, Sigma, St. Louis, MO, USA) was introduced to each well at a concentration of 1.0 μg/mL, and the cells were incubated for 24 h. Positive and negative controls were included where cells were incubated without LPS and compounds, and with LPS alone, respectively. Following that, the samples underwent centrifugation at 1000× *g* for 20 min, and the resulting supernatants were collected and stored on ice for future applications. The cells were gently washed with cold PBS and then resuspended in fresh lysis buffer (0.1 mL/well). Plates containing the samples were subsequently centrifuged at 1500× *g* for 10 min at 2–8 °C to eliminate any cellular debris. The mixture of supernatants and cell lysates was promptly subjected to analysis for TNF levels using the Mouse TNF-α ELISA Kit SEA133Mu TNF-α (Cloud-Clone Corp., Katy, TX, USA).

### 4.7. COX-2 Activity Assay Kit

RAW 264.7 cells were seeded in 96-well plates at a density of 2 × 10^4^ cells in a volume of 200 µL per well. The plates were incubated for 2 h at 37 °C in a 5% CO_2_ incubator to allow the cells to adhere. After the adhesion period, the cells were treated with compounds **U-443** or **U-573** at concentrations ranging from 0.1 to 1.0 µM for 1 h. Subsequently, LPS was added to each well at a concentration of 1.0 μg/mL, and the cells were incubated for 24 h. Positive and negative controls were included where cells were incubated without LPS and compounds and with LPS alone, respectively.

To prepare the cell lysate, the cells were washed once with 0.2 mL of PBS and resuspended in the same volume of PBS. Cells were then centrifuged at 500× *g* for 3 min. The cell pellet was resuspended in 0.1 mL of lysis buffer containing a protease inhibitor cocktail. After vortexing and incubating on ice for 5 min, the lysate was again centrifuged at 12,000× *g*, 4 °C for 3 min. The supernatant was collected and kept on ice for further use. The activity of cyclooxygenase-2 (COX-2) was determined using the Cyclooxygenase Activity Assay kit (BN00779, Assay Genie, Ireland) according to the manufacturer’s protocol.

### 4.8. Western Blotting

Western blotting was used to determine the expression of IL-1β and pro-IL-1β. RAW 264.7 cells were incubated with the test compounds at concentrations of 0.1 and 1.0 μM for 24 h. Afterward, LPS was added and the cells were further incubated for another 24 h. The cells were then washed with cold PBS (BioloT, Russia) and lysed using the RIPA buffer (Sigma-Aldrich, St Louis, MO, USA). The protein concentration was measured using the Bradford method. Electrophoresis with SDS on a 12% polyacrylamide gel was performed to separate the proteins. The separated proteins were transferred onto Immobilon®-P polyvinylidene difluoride (PVDF) membrane using a semi-dry transfer cell (Bio-Rad, Hercules, CA, USA). Specific primary polyclonal antibodies against IL-1β (Invitrogen, Waltham, MA, USA) (1:1000) were used to detect the IL-1β protein zones, while specific monoclonal antibodies against β-actin were used to detect the β-actin protein zones as a loading control. Horseradish peroxidase-conjugated (Sigma-Aldrich, St. Louis, MO, USA) secondary antibodies were used. Protein zones on the membrane were visualized using the Pierce^TM^ ECL kit (Thermo Fisher Scientific, Waltham, MA, USA) and a Versa Doc imaging system (Bio-Rad, Hercules, CA, USA). Densitometry analysis of the protein zones was carried out using Image Lab 6.0.1 software (Bio-Rad, Hercules, CA, USA).

### 4.9. Determination of Antioxidant Activity in Brain Homogenate

The brains of male CD1 mice were rinsed with a cold buffer solution containing 140 mM KCl and 10 mM K_2_HPO_4_ at pH 7.4. The brains were then homogenized using a Potter-Elvehjem tissue homogenizer, with the buffer added to the brain tissue at a weight ratio of 1:4. To the resulting homogenate, 10 mL of buffer was added, and the mixture was centrifuged for 10 min at 900 rpm using a Labofuge 44R centrifuge (Thermo Scientific, Germany). The supernatant was transferred to a volumetric flask and adjusted to a final volume of 25 mL with the buffer. The Lowry protein concentration in the homogenate was found to be within the range of 0.27–0.3 mg/mL.

To evaluate the antioxidant activity of the compounds in the absence of enzymatic reactions, 0.5 mL of distilled water or a solution of the tested compound in water was combined with 0.5 mL of brain homogenate. The mixture was then incubated for 10 min, followed by the addition of 20 μL of a 2 mM FeSO_4_ solution. The reaction was terminated by introducing 1 mL of 20% trichloroacetic acid (TCA, Reachim, Russia) into the system. Subsequently, the homogenate was subjected to centrifugation for 10 min at 4000 rpm using a Labofuge 44R centrifuge. To the 1 mL of supernatant, 1 mL of a freshly made solution of 0.7% thiobarbituric acid (TBA, Sigma, USA) in 50% glacial acetic acid was added. This mixture was then incubated for 30 min in a water bath at 100 °C to allow the reaction with TBA to occur. The resulting products that react with TBA were assessed by determining the content of TBA-reacting substances (TBARS) using a spectrophotometric method. A PHERAstar FS plate reader (MG Labtech, Germany) was utilized to measure the fluorescence intensity at wavelengths of 480/520 nm (excitation/emission). TBA-reacting products were determined in the initial homogenate of the mouse brain (spontaneous level, F_sp_), in the homogenate after incubation with an oxidizing agent (induced level, F_in_), and in the homogenate after incubation with an oxidizing agent and a natural compound in the experiment (experimental level, F_exp_). The content of TBARS after iron-induced oxidation was F_in_ − F_sp_ and was taken as 100%. The content of TBARS (%) after oxidation in the presence of compounds was (F_exp_ − F_sp_ )/(F_in_ − F_sp_) × 100%. Ionol (2,6-di-*tert-*butyl-4-methylphenol, Sigma-Aldrich, St Louis, MO, USA) was used as a reference antioxidant compound.

### 4.10. Molecular Modeling

The spatial structures of 1,4-NQs used in the work were obtained and optimized using the potential of the forces MFF94 using the MOE program [56]. The crystal structure of mouse cyclooxygenase-2 (mCOX-2) PDB code 1CX2 [44] was used for 1,4-NQ molecular docking. Molecular docking was performed with the docking module of the MOE program using a selective inhibitor SC-558 in a complex with mCOX-2 (PDB code 1CX2) as a template for the binding site. For each compound, 30 complexes were calculated using the London dG parameters, for 5 of which the optimization was performed with the GBVI/WSA dG parameters. The analysis of contacts in the complexes was carried out using the ligand interaction module. The calculations were performed using the Center for Collective Use of the IAPU FEB RAS "Far Eastern Computational Resource".

### 4.11. Animals

Experiments involving female CD1 mice aged two months (weighing 20–22 g) were carried out using mice obtained from the Russian National Center for Genetic Resources of Laboratory Animals. The mice were housed in a specific pathogen-free (SPF) vivarium at the ICiG SB RAS in Novosibirsk, Russia. They were provided with food and water in an environment with a 12 h light/dark cycle at a temperature of 22 ± 1 °C. All experimental procedures adhered to the International Recommendations for Biomedical Research Using Animals established by the International Council of Medical Scientific Societies (CIOMS), as well as the Laboratory Practice Rules approved by the Ministry of Health and Social Development of Russia on 23.08.2010 (Order No. 708n) and approved by the local ethics committee of the G.B. Elyakov Pacific Institute of Bioorganic Chemistry, Far Eastern Branch of the Russian Academy of Sciences (protocol No 01/20 of September 6, 2020).

### 4.12. Parkinson Disease Induction

The early stage of Parkinson’s disease was induced in mice according to [10,57] with modifications. Rotenone (Sigma-Aldrich, St Louis, MO, USA) was dissolved in dimethyl sulfoxide and olive oil (1:9 *v*/*v*). Dissolved rotenone was injected subcutaneously during 8 consecutive days with a dose of 6 mg/kg to construct the model.

L-DOPA (3,4-dihydroxy-L-phenylalanine, Sigma-Aldrich, St Louis, MO, USA) was used as a positive control in an experiment at a dose of 5 mg/kg per oral, once, one day after the last dose of rotenone. After 40 min, the effect of L-DOPA was assessed.

Studied compounds **U-443** and **U-573** were dissolved in dimethyl sulfoxide and olive oil (1:9 *v*/*v*). Compounds were administrated intraperitoneally at doses 0.1, 1.0, or 10.0 mg/kg, three times every second day. The solvent dimethyl sulfoxide and olive oil (1:9 *v*/*v*) were used instead of naphthoquinone in the control group of mice treated with rotenone only.

### 4.13. Experimental Procedure

The experimental procedure proceeded as outlined below. Once the mice had acclimated, they were randomly divided into six groups: the first group consisted of control mice in their normal state; the second group served as the control for the model (receiving a solvent); the third group received L-DOPA at a dosage of 5 mg/kg; and the fourth to sixth groups constituted the model groups exposed to 1,4-naphthoquinones at dosages of 0.1, 1.0, or 10.0 mg/kg. Behavioral assessments were conducted one day after the final injection using the “Cylinder” and “Open field” tests.

#### 4.14. “Cylinder” Test

The transparent glass cylinder, measuring 19.5 cm in height and 15.0 cm in diameter, was partially covered with black cardboard on three sides to minimize the impact of ambient lighting. The side of the cylinder facing the camera was left uncovered for video recording purposes. The camera SONY (Carl Zeiss, VirioTessar, 70×) was used during the experiment. Mouse behavior was recorded for 3 min. To prevent any disturbances in the behavior of the mice, measures were taken to eliminate noise and minimize changes in light during the process. Afterward, the cylinder was thoroughly cleaned using water, followed by sanitization of the inner wall using 70% ethanol to eliminate mouse scents. It was then dried before introducing another mouse. The changes in the behavioral reactions of mice were assessed against the background of the development of the rotenone model. The number of mouse climbs (rearings) was calculated. A rearing behavior in mice is characterized by the act of the mouse standing on its hind limbs, lifting both forelimbs above shoulder level, and subsequently descending. The elevation of a single front limb is not considered in this test.

#### 4.15. “Open field” Test

For the open field test, a green opaque plastic box measuring 100 × 100 × 70 cm was employed. Prior to the assessment, a preadaptation period of 5 min was allotted for all mice within the box. Subsequently, a pair of mice was gently placed in the center of the open field, and their movements were recorded for a duration of 3 min using a digital camera (SONY, Carl Zeiss, VirioTessar, 70×). To assess changes in animal behavior, the “ToxTrac” v.2.84 software (UMEA University, LinneaausVag, SE-901 87, Sweden) was used for data collection, plotting, and data analysis. The average speed (sm/min), total distance (mm), total time frozen (min), and frozen events (number of times) were determined. Between each trial, the box was thoroughly cleaned using a solution of 10% ethanol and water.

### 4.16. Acute Toxicity

To determine acute toxicity of 1,4-NQs CD1, female mice, weighing 20–22 g, 6 animals per group, were used. The mice were kept on a standard vivarium diet. The substances were diluted with olive oil and injected intraperitoneally into the mice in a volume of 0.1 mL. The control and experimental animals were observed for two weeks.

The acute toxicity was assessed using the Kerber formula [58]:

LD_50_ = LD_100_ – Σ (z × d)/m;

where LD_50_ is the death rate of 50% of mice; LD_100_ is the death rate of all animals; z is a half of the sum of the animal number that died from the last two doses; d is the interval between every two last doses; m is the number of animals for each dose.

### 4.17. Data Analysis and Statistics

The data were collected in three separate replicates, and the calculated values were presented as mean ± standard error of the mean (SEM). Statistical analysis was performed using SigmaPlot 14.0 (Systat Software Inc., San Jose, CA, USA), utilizing one-way analysis of variance (ANOVA) followed by the post hoc Student–Newman–Keuls test.

## 5. Conclusions

Thus, the two 1,4-naphthoquinone derivatives we synthesized, **U-443** and **U-573**, demonstrated moderate cytoprotective properties in two murine cell lines, neuroblastoma Neuro-2a and macrophages RAW 264.7, which mimic mouse brain neurons and microglia, correspondingly. We have shown that the studied 1,4-NQs are capable of not very pronounced but reliable and significant protection of neuronal and macrophage cells from the cytotoxic impact of the neurotoxin rotenone. The protecting effect was accompanied by the ability of 1,4-NQs in the noncytotoxic nanomolar concentration range to modestly reduce the production of ROS and restore the MMP of neuronal cells in the presence of neurotoxin, indicating some preservation and recovery of cell physiology, as well as the ability to slightly but significantly diminish the content of such proinflammatory markers as ROS, NO, TNF and IL-1β, whose elevation was induced in LPS- or rotenone-inflamed macrophages.

Possibly, in addition to the registered effects, the protective and anti-inflammatory properties of the studied 1,4-NQs may be associated with their noticeable antioxidant activity and the ability to markedly inhibit the COX-2 enzyme in LPS-treated cells, resulting in partial elimination of neuroinflammation. Potentially, all these features of the mechanism of action of the studied naphthoquinones contribute to the manifestation of observed acceptable neuroprotective effects in animals with rotenone-induced parkinsonism.

At the same time, one of the studied compounds, **U-443**, exhibits certain cytotoxic activity, which precludes its further study as a cytoprotector. The substance **U-573** seems to us more promising. The new knowledge obtained in the present study provides the basis for future-directed organic synthesis of new 1,4-NQs derivatives with improved on-target efficacy and lack of toxicity using this small molecule as a scaffold.

## Data Availability

The original data are available from the corresponding author on request.

## Supplementary Materials

The following supporting information can be downloaded at: https://www.mdpi.com/article/10.3390/md22020062/s1, Figure S1: ^1^H and ^13^C NMR spectra of compound **4 (U-573)**; Figure S2: ^1^H and ^13^C NMR spectra of compound **8 (U-443)**; Figure S3: HPLC chromatogram of compound **4 (U-573)**; Figure S4: HPLC chromatogram of compound **8 (U-443).**
