# Supplementary material for: Protection Activity of 1,4-Naphthoquinones in Rotenone-Induced Models of Neurotoxicity
Source: Mar Drugs. 2024 Jan 25;22(2):62. doi: 10.3390/md22020062 (PMC10890484; doi:10.3390/md22020062)
Supplement: Supplementary file 1 [file marinedrugs-22-00062-s001.zip › marinedrugs-2784877-supplementary.pdf]

Supplementary Material

Protection activity of 1,4-naphthoquinones in rotenone-induced models of neurotoxicity.

Irina Agafonova[1§], Ekaterina Chingizova[1§], Elena Chaikina[1], Ekaterina Menchinskaya[1], Sergey Kozlovskiy[1], Galina Likhatskaya[1], Yuri Sabutski[1], Sergey Polonik[1], Dmitry Aminin[1,2] and Evgeny Pislyagin[1*]

[1] G.B. Elyakov Pacific Institute of Bioorganic Chemistry, Far-Eastern Branch of the Russian Academy of Science, 690022 Vladivostok, Russia

[2] Department of Biomedical Science and Environmental Biology, Kaohsiung Medical University, No. 100, Shin-Chuan 1st Road, Sanmin District, Kaohsiung City 80708, Taiwan

[§] Equal contribution

* Correspondence: pislyagin@hotmail.com (EP); Tel.: +7(4232)319932

Table of Contents

Figure S1. ^{1}H and ^{13}C NMR spectra of compound **4** **(U-573)**

Figure S2. ^{1}H and ^{13}C NMR spectra of compound **8** **(U-443)**

Figure S3. HPLC chromatogram of compound **4** **(U-573)**

Figure S4. HPLC chromatogram of compound **8** **(U-443)**

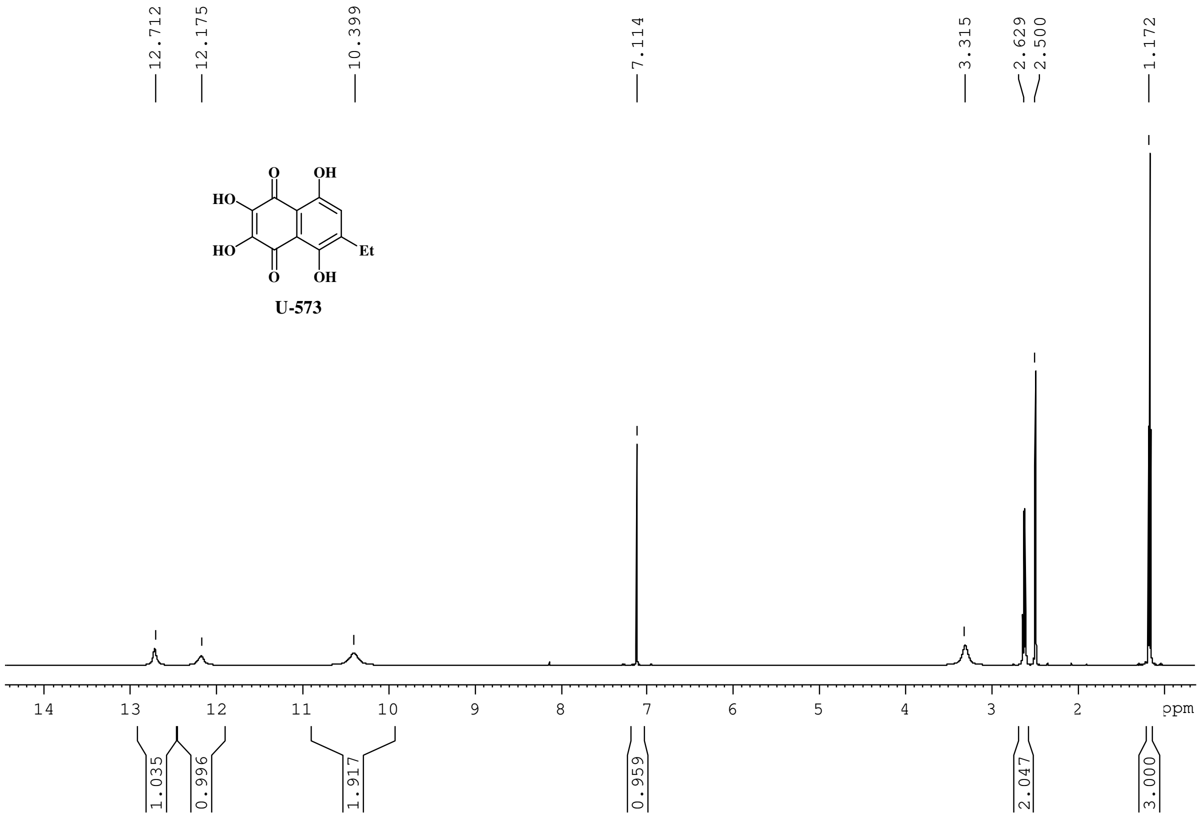

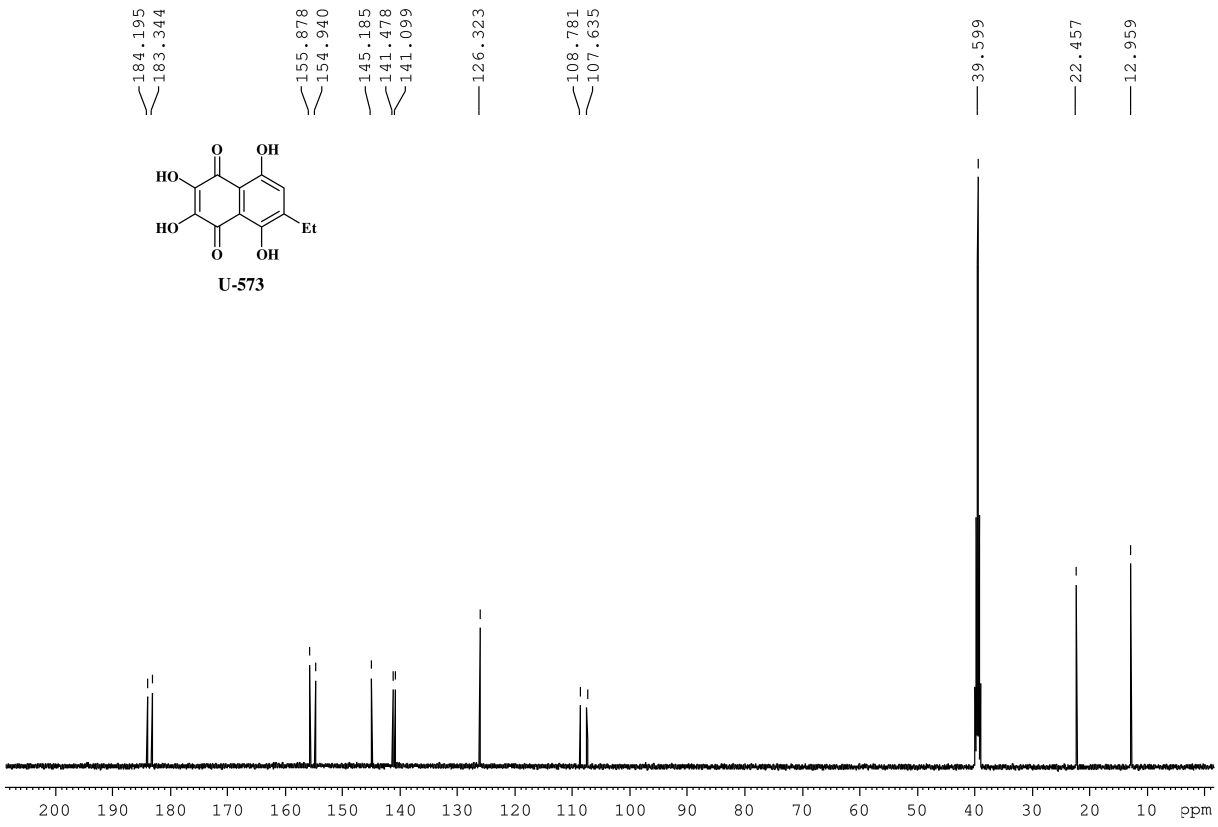

Figure S1. ^{1}H and ^{13}C NMR spectra of compound **4** (**U-573**) (solvent - $DMSO_{d-6}$)

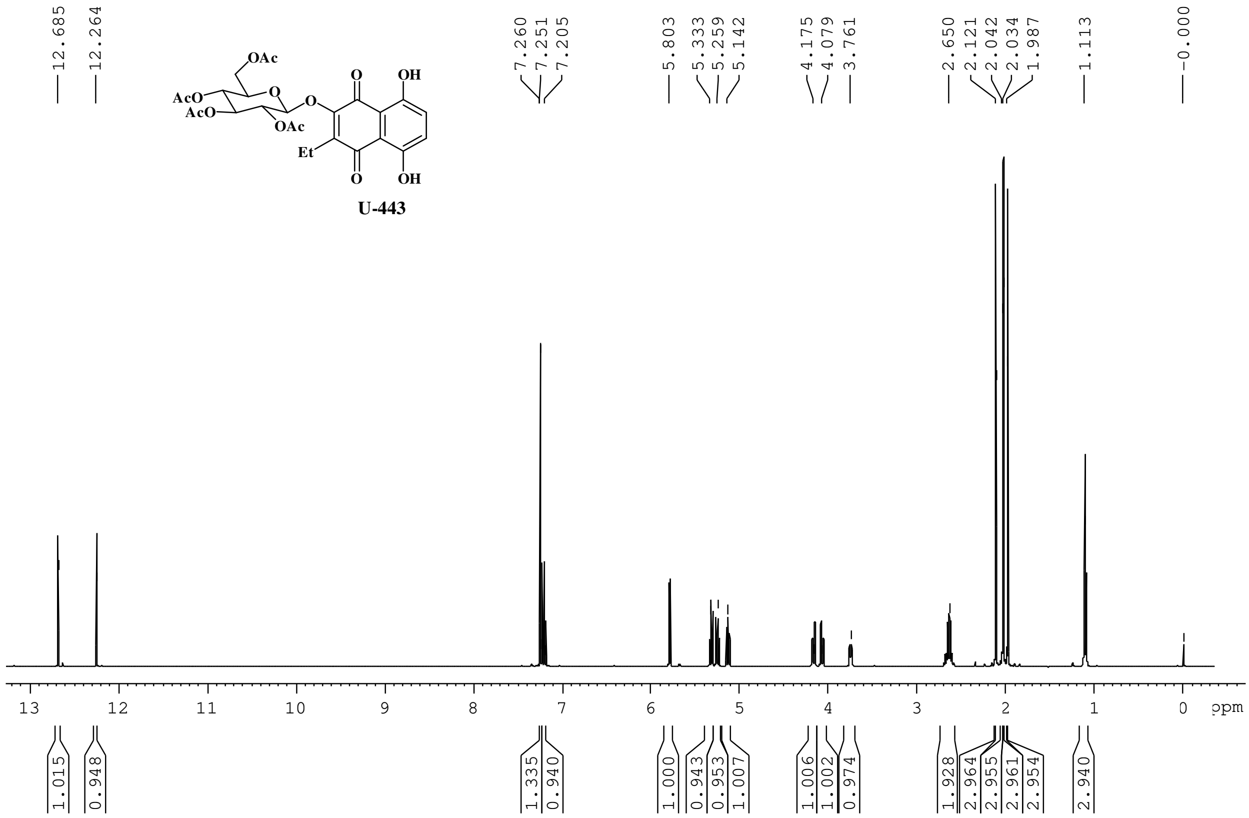

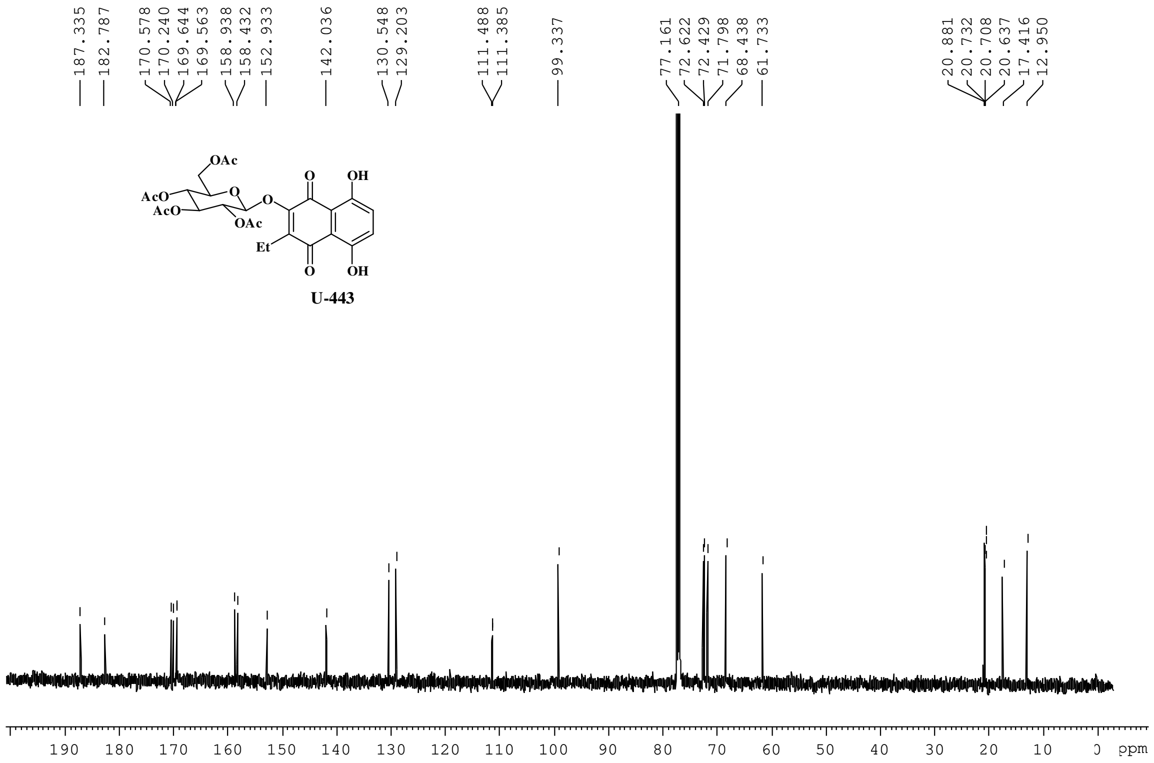

Figure S2. ^{1}H and ^{13}C NMR spectra of compound **8** (**U-443**) (solvent – $CDCl_3$)

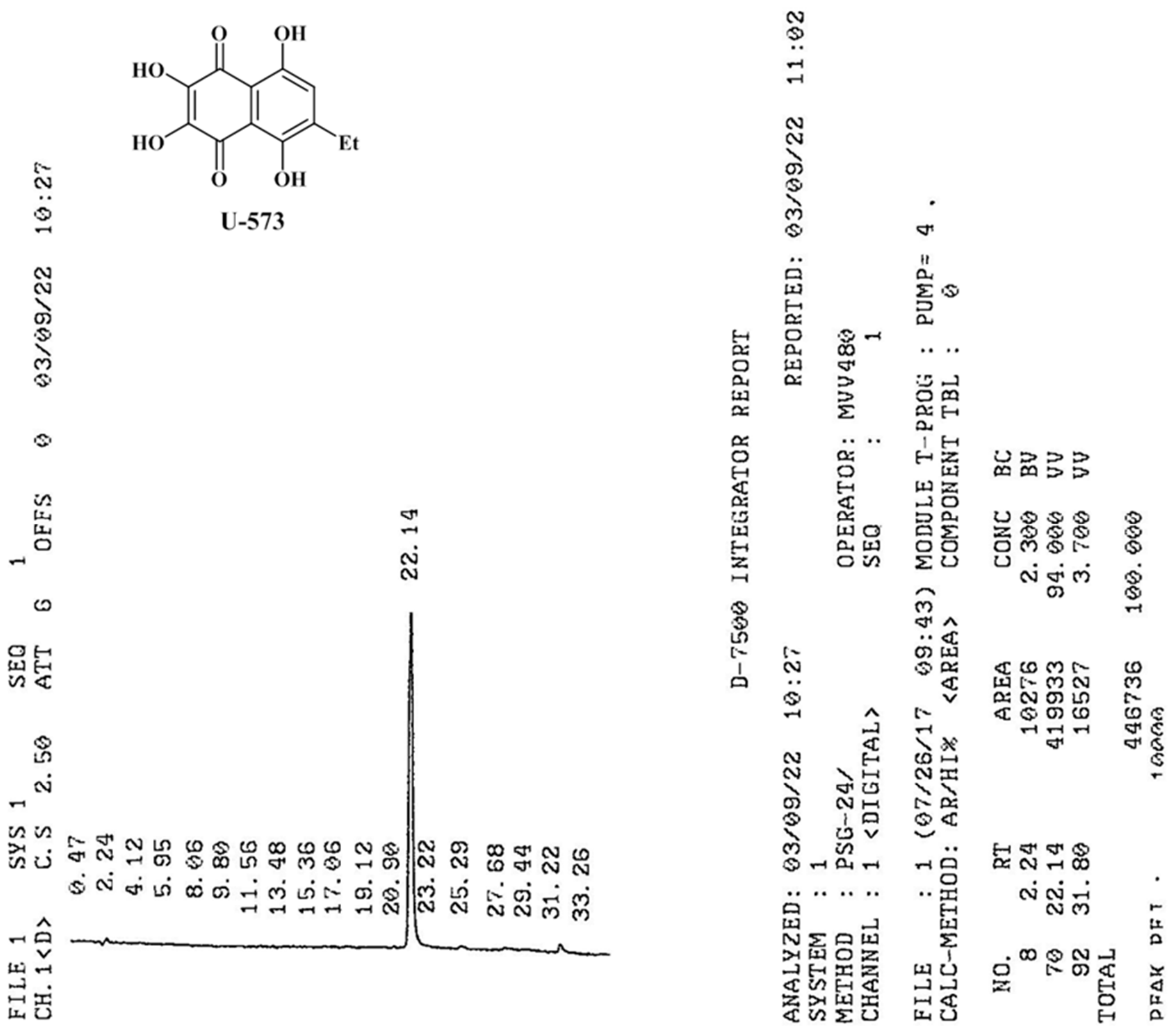

Figure S3. HPLC chromatogram of compound **4** (**U-573**)

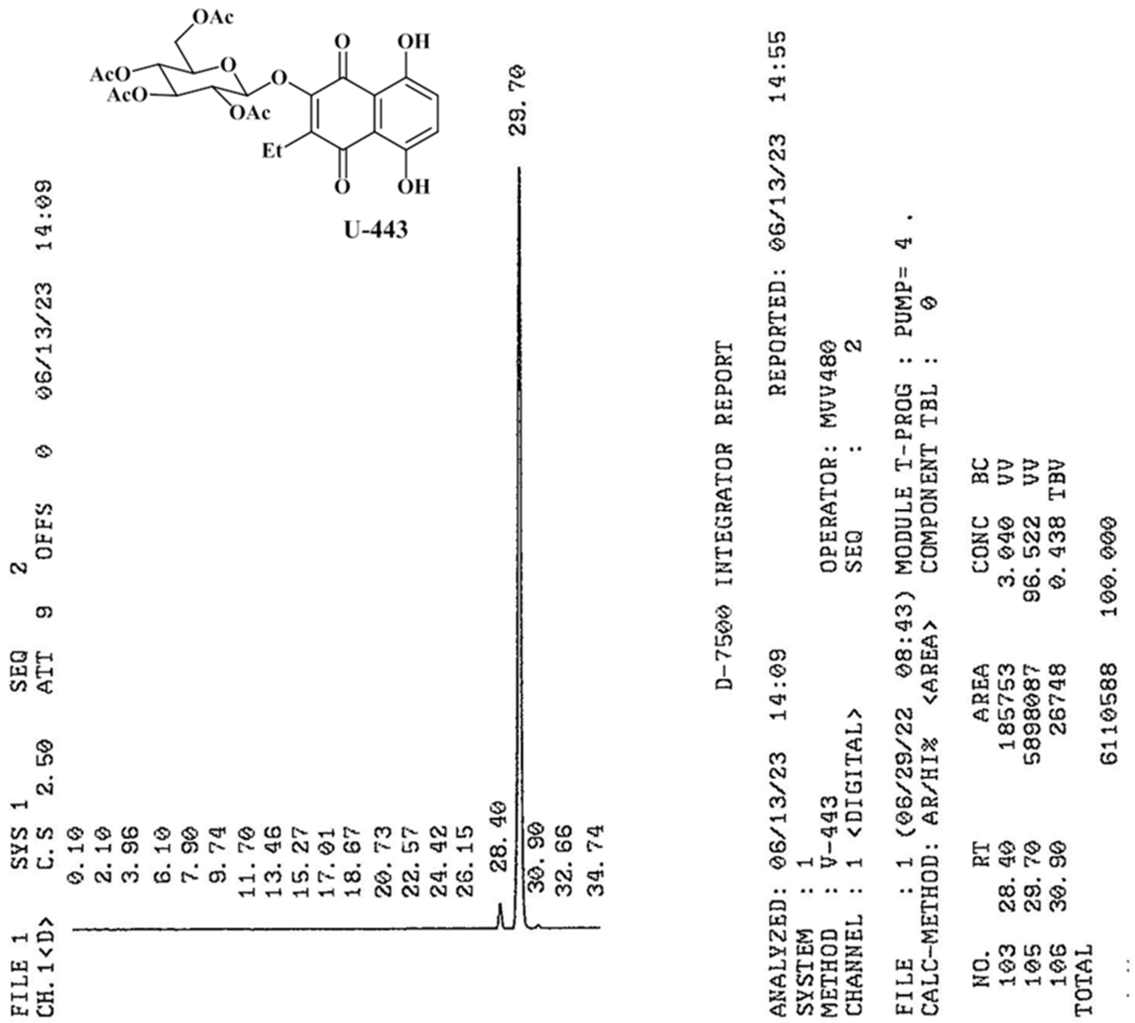

Figure S4. HPLC chromatogram of compound **8 (U-443)**
